# Biomaterial and Therapeutic Approaches for the Manipulation of Macrophage Phenotype in Peripheral and Central Nerve Repair

**DOI:** 10.3390/pharmaceutics13122161

**Published:** 2021-12-15

**Authors:** Adrian Dervan, Antonio Franchi, Francisco R. Almeida-Gonzalez, Jennifer K. Dowling, Ohemaa B. Kwakyi, Claire E. McCoy, Fergal J. O’Brien, Alan Hibbitts

**Affiliations:** 1 Tissue Engineering Research Group, Department of Anatomy and Regenerative Medicine, Royal College of Surgeons in Ireland, D02 YN77 Dublin, Ireland; adriandervan@rcsi.ie (A.D.); antoniofranchi@rcsi.ie (A.F.); franciscoalmeida@rcsi.ie (F.R.A.-G.); fjobrien@rcsi.ie (F.J.O.); 2 Trinity Centre for Bioengineering, Trinity College Dublin, D02 R590 Dublin, Ireland; 3 Advanced Materials and Bioengineering Research Centre (AMBER), Royal College of Surgeons in Ireland and Trinity College Dublin, D02 YN77 Dublin, Ireland; 4 School of Pharmacy and Biomolecular Sciences, Royal College of Surgeons in Ireland, D02 YN77 Dublin, Ireland; jenniferdowling@rcsi.ie (J.K.D.); OhemaaKwakyi@rcsi.ie (O.B.K.); clairemccoy@rcsi.ie (C.E.M.); 5 FutureNeuro SFI Research Centre, Royal College of Surgeons in Ireland, D02 YN77 Dublin, Ireland; 6 School of Medicine, Royal College of Surgeons in Ireland, D02 YN77 Dublin, Ireland

**Keywords:** macrophage, inflammation, peripheral nerve, central nervous system, biomaterials, regenerative medicine, immunology

## Abstract

Injury to the peripheral or central nervous systems often results in extensive loss of motor and sensory function that can greatly diminish quality of life. In both cases, macrophage infiltration into the injury site plays an integral role in the host tissue inflammatory response. In particular, the temporally related transition of macrophage phenotype between the M1/M2 inflammatory/repair states is critical for successful tissue repair. In recent years, biomaterial implants have emerged as a novel approach to bridge lesion sites and provide a growth-inductive environment for regenerating axons. This has more recently seen these two areas of research increasingly intersecting in the creation of ‘immune-modulatory’ biomaterials. These synthetic or naturally derived materials are fabricated to drive macrophages towards a pro-repair phenotype. This review considers the macrophage-mediated inflammatory events that occur following nervous tissue injury and outlines the latest developments in biomaterial-based strategies to influence macrophage phenotype and enhance repair.

## 1. Introduction

**Figure 1:**
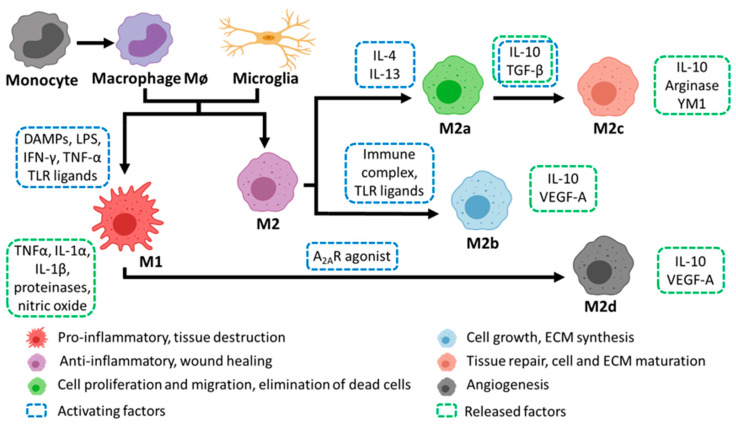
Schematic diagram of macrophage polarization after nerve injury. Macrophages constantly undergo phenotypic polarization depending on the current environmental stimuli. M1 macrophages are activated by TLR ligands, LPS, TNFα, IFN-γ. Activation into M2 macrophages occurs when macrophages are exposed to IL-4, IL-13, TGF-β, immune complexes and A2AR agonist. (Adapted from [3,4], Wolters Kluwer, 2019 and Springer Berlin Heidelberg, 2015. Created with Biorender^®^).

Macrophages are innate immune cells and play a critical role in modulating injury and potential repair in the peripheral nervous system (PNS) and the central nervous system (CNS). Together with the neuronal-specific immune cells, the microglia, macrophages can undergo phenotypic shifts into either a pro-inflammatory (M1) or a range pro-repair (M2) sub-types (Figure 1) [1]. Phenotypic transitions between non-polarized macrophages to M1/M2 phenotypes are guided by external stimuli, particularly stimulation of toll-like receptors (TLRs) and secreted cytokines from injured and neighboring tissue [2]. Furthermore, the polarization state is time dependent and typified by a sequential transition from M1 to M2 phenotype(s) as the tissue response to injury progresses. However, the response differs markedly in PNS compared to the dysfunctional CNS repair processes. Accumulating evidence over the past number of years suggests that the various features of biomaterials, such as composition, and physical and chemical modifications, plus the inclusion of therapeutic molecules, cell and gene therapies, all can have a direct effect on macrophage numbers and their polarization in the injured nervous system. Through a systematic approach we outline recent developments in the use of biomaterials that not only support and promote axonal growth but also help modulate macrophage polarization in both PNS and CNS tissues to enhance regeneration.

## 2. Macrophages in Peripheral Nervous System Injury and Repair

### 2.1. Overview of Peripheral Nerveous System (PNS) Injury 

The PNS has a remarkable capacity for repair. Damaged axons are capable of spontaneous, supported regeneration following injury, and can restore functional motor and sensory connections to distal targets depending on the defect length. A series of orchestrated events occur to mend the affected axons and recover neural functions after a traumatic peripheral nerve injury (PNI). This repair process is divided into three sequential stages encompassing 1. Wallerian degeneration, 2. axonal regrowth and 3. restoration of nerve function [5]. 

#### 2.1.1. Distal Defect Wallerian Degeneration

Wallerian degeneration is mediated by an initial innate-immune response after PNI. This involves the activation, migration, proliferation and differentiation of Schwann cells (which possess glial-like functions within the PNS), fibroblasts and macrophages to degrade damaged axons and prepare the environment for regrowth [6]. Firstly an efflux of calcium from the mitochondria and endoplasmic reticulum infiltrate the site of injury, which leads to the activation of the ion-sensitive protease calpain [7]. This protease, in combination with the ubiquitin-proteasome system [8], starts degrading the distal axon segments into granules. At the distal segment of the injured nerve, resident Schwann cells detach from the damaged axons, resulting in demyelination, leaving the axon unprotected, and the detached myelin sheaths become segmented longitudinally, acquiring an ovoidal-like shape [9]. It takes approximately 48 h for Schwann cells to stop the production of myelin lipids and proteins [10]. Although axon degeneration can be detected after 36–44 h in mice, in humans, detection of fragmented axons (assessed through nerve excitability tests) can take from 4 to 10 days [10,11]. 

Over the course of nerve repair in the proximal and distal injured nerve segments PNS, the Schwann cell population change their phenotype to progenitor-like Schwann cells and start overexpressing pro-inflammatory cytokines and chemokines to initiate macrophage activity. At the same time, the blood–nerve barrier, mainly composed of endothelial cells [12], breaks apart along the entire nerve. This facilitates the infiltration of B cells [13], resident macrophages and recruited monocyte-derived macrophages, amplifying the inflammatory response. It is worth noting that the degenerated myelin and granulated axonal debris contains myelin-associated glycoprotein (MAG) and oligodendrocyte-myelin glycoprotein (OMgp), which are highly cytotoxic and hamper axonal regrowth [14,15]. B lymphocytes secrete opsonins, coating and labeling the granulated axons and degraded myelin, facilitating the phagocytic activity of macrophages [16]. Therefore, in the first 3 days post-injury, progenitor-like Schwann cells and macrophages continuously phagocytose and eliminate debris and prepare the site for axonal regrowth [6].

#### 2.1.2. Axonal Regrowth

At approximately 7–10 days post-injury, the axonal regrowth phase starts [6]. Progenitor-like Schwann cells start organizing into multicellular cylindrical conduits, or bands of Büngner, secreting laminin-2, laminin-8, growth factors and trophic factors (Glial cell-derived neurotrophic factor (GDNF), Brain Derived Neurotrophic Factor (BDNF), Neurotrophin-3 (NT3), Nerve Growth Factor (NGF), Vascular Endothelial Growth Factor (VEGF), and pleiotrophin) to mature and guide the regenerating axons to the proximal nerve stump [9,17]. Furthermore, the Schwann cells at the distal stump upregulate the expression of cytokines including Tumor Necrosis Factor (TNF)-a, LIF, Interleukin (IL)-1a, IL-1b, Leukemia inhibitory factor (LIF), and Monocyte chemoattractant protein-1 (MCP-1), which serve to recruit macrophages [18]. Depending on the type of injury, two scenarios are possible. Typically, when the nerve is crushed, the basal lamina around the Schwann cells is preserved and therefore the axonal pathway is not lost. Consequently, axons can regrow through the bands of Büngner following their former pathways with high efficiency.

In instances where there is nerve transection, a tissue bridge of macrophages, neutrophils, fibroblasts, endothelial cells, fibronectin and elastin must be built between the gap. This cellular bridge serves as a ‘travel path’ for Schwann cells and axons [19,20]. At this point, macrophages are the most abundant cell type populating the injury site, secreting vascular endothelial growth factor (VEGF-A) to promote polarized angiogenesis on both ends of the transected nerve. This leads to a more oxygenated environment around the damaged nerve where Schwann cells and axons can migrate and regrowth can occur. In addition, collagen I, collagen IV and laminin can be found around the blood vessels, which are known to promote cell growth and attachment through integrins between Schwann cells and the vascularized tissue bridge [19,21]. As a result, these bands of Büngner follow the newly formed vessels and guide the regenerating axons across the nerve bridge [22]. Importantly, severing of blood vessels during this stage results in defective nerve repair. This stage ends when progenitor-like Schwann cells migrate from both sides of the injury gap, closing the defect site allowing axons to cross from the distal to the proximal site of the nerve [19].

#### 2.1.3. Restoration of Nerve Function

In the final phase, which can take approximately 20 days [6], the inflammation and macrophage numbers reduce with some of the recruited macrophages remaining in place as resident macrophages to further facilitate repair. Any surplus macrophages will leave the region and return to the lymphatic organs or spontaneously undergo apoptosis [23]. At the same time, the blood–nerve barrier gradually regains its protective function [24]. As axons begin to reform functional connections with their distal targets, Schwann cells differentiate into a myelinated phenotype producing laminin-2, which is critical for re-myelinating and sheathing the axon. This facilitates the transmission of electric impulses efficiently and finally restores nerve function [25]. 

### 2.2. Macrophage Recruitment during PNI 

Although Schwann cells are capable of proliferating and degrading the detached myelin and debris without the help of macrophages [26], the surrounding microenvironment gradually becomes hypoxic (pO_2_ < 10 mmHg) and necrotic [3]. Activated Schwann cells cannot phagocytize debris in this environment over the long term without additional support. To attract other phagocytosing cells, Schwann cells secrete collagen VI, which acts as a chemoattractant and upregulates the production of pro-inflammatory cytokines (TNF-α, IFN-γ, IL-1α and IL-1β) [27]. This also induces fibroblasts to secrete the cytokines IL-6, granulocyte-macrophage colony-stimulating factor (GM-CSF) and leukocyte inhibitory factor (LIF) [28,29]. Furthermore, Schwann cells, fibroblasts, and endothelial cells start producing pancreatitis-associated protein (PAP-III), monocyte chemoattractant protein-1 (MCP-1/CCL2), and macrophage inflammatory protein-1 (MIP-1/CCL3) chemokines, which encourage the migration of neutrophils, monocytes and resident macrophages into the injured nerve [4,30].

Neutrophils, capable of resisting the low oxygen environment rapidly infiltrate the lesion site and briefly help in clearing the debris before undergoing apoptosis within 3 days after injury [31]. Resident macrophages, which normally act as an immune surveillance system, start proliferating and migrate to the injury site [32], while also releasing chemokines and cytokines for the continued recruitment of circulating monocytes, which are then differentiated into macrophages [13]. Recruited monocyte-derived macrophages release the chemokines MCP-1/CCL2, MIP-1/CCL3 and CCL7 to further perpetuate monocyte infiltration. Studies in animal/human PNI models have shown that after 7 days the average ratio of infiltrated monocyte-derived macrophages to resident macrophages is 3:1 [32]. However, more recent studies have demonstrated that the population of macrophages at the site of injury consist of up to 90% recruited monocyte-derived macrophages [33,34]. This mixed population of resident and recruited macrophages, in combination with Schwann cells, continues to phagocytose debris.

### 2.3. Macrophage Polarization and Roles during PNI

**Figure 2:**
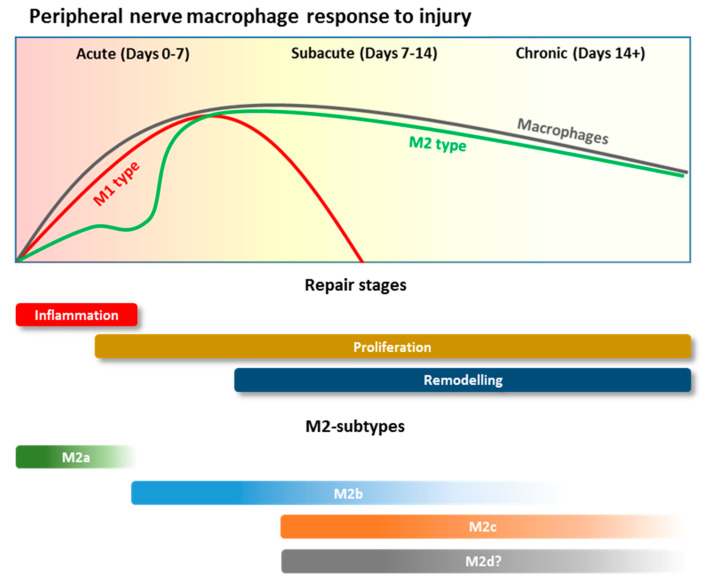
Temporal changes in macrophage phenotype during PNI and repair.

Macrophage polarization plays a critical role in peripheral nerve repair process. The current viewpoint is that during and after PNI macrophages possess the ability to exist along a spectrum of polarization states (Figure 2). After the initial influx of peripheral and systemic macrophages both M1 and M2 phenotypes are present in the injury site, although the number of M1 cells greatly exceeds their M2 counterparts. This however, is followed by a progressive and steady transition from an M1 to M2 polarized state after 3 to 5 days [35].

M1 macrophages are typically activated by toll-like receptor (TLR) ligands, such as lipopolysaccharide (LPS) or the cytokines, tumor necrosis factor alpha (TNF-α) and interferon gamma (IFN-γ) [33]. However in PNI, damage associated molecular patterns (DAMPs) such as high mobility group box-1 (HMGB-1) and debris released by injured neurons and Schwann cells has been shown to activate the M1 state in macrophages and infiltrating monocytes [36]. Most notably in PNI, macrophages sense and respond to the hypoxic environment by activating the transcription factor, hypoxic inducible factor 1 alpha (HIF-1α) [37]. This in turn stimulates angiogenesis via activation of vascular endothelial growth factors (VEGFs) promoting blood vessel repair and growth, in addition to a route for the migration of Schwann cells to the injury site. M1 macrophages are also prominent phagocytes at the injury site, playing a critical role in removing debris, phagocytosis of apoptotic neutrophils, growth factor production, and remodeling of the extracellular matrix (ECM) of the distal nerve in the initial days post injury [38]. However, a prolonged and chronic presence of M1 macrophages can be detrimental to repair [39]. M1 macrophages typically display a cytotoxic and pro-inflammatory phenotype, releasing proteinases, nitric oxide, and free radicals, worsening the inflammation and tissue destruction [40,41].

Importantly, following the initial pro-inflammatory response in PNI, there is a transition to an anti-inflammatory or M2 macrophage phenotype that begins to occur in the nerve 3 days post-injury [42]. Alternative activation into the M2 macrophage phenotype occurs when macrophages are exposed to interleukins (ILs) such as IL-4, IL-13 or transforming growth factor beta (TGF-β), immune complexes and adenosine A_2A_ receptor agonists. [4]. Additionally, while Schwann cells do not secrete M2 polarizing cytokines such as IL-4, IL-10 or IL-13, they are potent inducers of M2 macrophages [43]. In vitro studies have found that macrophages and Schwann cells co-localize and remain in contact followed by elongation of macrophages and their expression of CD163. The exact mechanism of polarization remains under investigation [44].

As M2 macrophages populate the lesion site they release a series of anti-inflammatory cytokines, regulating and balancing the pro-inflammatory response of the M1 macrophages to maintain homeostasis and mediate tissue regeneration [1]. M2 macrophages are characterized by markers including arginase-1 (Arg1), chitinase-like 3/YM1 (Ym1) and mannose receptor C type 1 (CD206/Mrc1). They also release IL-10, which is important in reducing the inflammatory response and inducing wound healing [45]. In addition, M2 macrophages can further change their profiles in response to local micro-environmental signals into four sub-types with very specific roles. The first subtype, M2a macrophages, remove dead cells and secrete anti-inflammatory cytokines and growth factors that promote cell proliferation and migration (IL-10, TGF-β). M2b macrophages promote cell growth and extracellular matrix (ECM) synthesis, releasing IL-10 and VEGF-A. The presence of IL-10 and TGF-β, secreted by M2a macrophages, polarizes cells to the M2c subtype. These are responsible for enhancing tissue repair, cell and ECM remodeling (via upregulation of matrix metalloproteinase-7 (MMP7), MMP8, and tissue inhibitor of metalloprotease 1 (TIMP1) [46]) by releasing Arginase and Ym1 [47]. Finally, M2d macrophages have an important role restoring blood flow, through the induction of polarized angiogenesis, as they secrete higher levels of VEGF-A, if compared to M2b macrophages [48].

Interestingly, M1 macrophages are the predominant phenotype in the lesion site 1-day post-injury; however, studies demonstrate the most highly upregulated genes are those that encode for enzymes, such as Arg1 and Chil3, which are associated with M2-type cells [49]. This emphasizes the fluid nature of macrophage sub-types and reinforces the theory that successful nerve regeneration is orchestrated by the co-existence and co-action of cells that exist along a spectrum of M1 and M2 activation states. The inflammatory process is further regulated by the arrival of T lymphocytes at the injury site. In rat models, these have been found to be evident at the injury site from as early as day 3 and peak in concentration at day 21 [50]. On arrival, they aid macrophages in the release of pro (TNF-α, IFN-γ) or anti-inflammatory (IL-4, IL-10) cytokines [51]. This leads to a reduction in the immune response, through downregulation of pro-inflammatory cytokine production, and signaling the end of macrophage-mediated inflammation in the nerve repair process [4]. 

## 3. Macrophages in Central Nervous System Damage and Repair

### 3.1. Overview of Central Nerve System (CNS) Injury

In marked contrast to the PNS, the CNS once injured, fails to mount a robust regenerative response and few if any axons from injured neurons regrow into the lesion site [52]. Instead a lesion cavity forms surrounded by a penumbra of injury responsive glial cells, comprising activated and reactive astrocytes, microglia and oligodendrocyte precursor cells [53]. The dynamics of macrophage infiltration and population of the lesion site early in the injury response mirrors the events seen in the PNS. Resident tissue macrophages and infiltrating blood borne monocytes infiltrate the lesion site to phagocytose debris and propagate the inflammatory response. However, this is where the similarity ends, as CNS lesion resident macrophages contribute to the scar forming process [54] and remain within the lesion cavity as it matures over many months [55,56]. These events occur in a highly organized fashion as described below.

CNS injury can arise from traumatic brain injury (TBI), ischemic stroke, Alzheimer’s disease, Parkinson’s disease, multiple sclerosis and motoneuron disease. By far, damage to the CNS through spinal cord injury is the most recognizable. Therefore, the following sections are focused primarily on events that occur in the lesioned spinal cord but have broader relevance to TBI and ischemia, and where possible these other injury types are also explored.

#### 3.1.1. Axon Degeneration

Following CNS injury, and in particular spinal cord injury, severed axons retract from the injury site, with the distal axon segment, similar to that found after PNI, slowly undergoing Wallerian degeneration over weeks to months [57] and the proximal axon retracting more rapidly over a period of hours, involving a neuron intrinsic process termed axon-dieback [58]. However, in the subsequent days after injury a second significant retraction occurs in proximal axons that is correlated with macrophage entry into the damaged spinal cord [59]. In a process that has been explored both in vitro [60] and in vivo [59], invading macrophages induce the secondary dieback of injured axons and their retraction bulbs through direct physical contact [60], specific ligand/receptor interactions [61,62,63] and protease secretion [61]. Experimental depletion of macrophages prevents axon-dieback in vivo [64] and even though activated microglia can become macrophagic and are often closely associated with axon retraction bulbs, they do not appear to be as neurotoxic [61]. Time-lapse multi-photon imaging of bone marrow chimera Cx3cr1+/GFP labeled macrophages elegantly demonstrated that monocyte-derived macrophages, rather than activated microglia, are responsible for secondary axonal damage after spinal cord injury (SCI) [59].

#### 3.1.2. Fibroglial Scar Formation

As inflammatory cells migrate into the lesion site, glial cells in and around the damaged region move to proliferate and seal off the developing lesion core through formation of a glial scar. Consisting of a complex mixture of cells and deposited growth inhibiting extracellular matrix, it generates a potent barrier between implanted biomaterials and injured axons [65,66]. Fibroglial scar formation is driven primarily by astrocytes, which begin entering a reactive state within hours of injury (astrogliosis) and typified by phenotypic changes that include hypertrophy and extension of thick processes together with increased glial fibrillary acidic protein (GFAP) immunoreactivity [67] and genotypic changes that polarize the cells into reactive subtypes [68]. At the lesion margins a subset of responsive astrocytes quickly divide and orient their processes perpendicular to the longitudinal axis of the cord and interdigitate to form a glia limitans barrier, typically found at the CNS periphery that attenuates macrophage influx into the injured cord [69]. Responsive astrocytes can also initiate inflammation by promoting the entry of inflammatory monocytes [69] and express receptors to a variety of pro-inflammatory chemokines, cytokines and DAMPs [70]. Astrocytes are further activated by expressing receptors to inflammatory molecules released by macrophages to induce a neurotoxic astrocyte phenotype and extensive astrogliosis [54,68,71]. 

Macrophages also play an important role in the formation of the secondary component of the scar tissue that develops in close apposition to the glia limitans. Infiltrating fibroblasts from meningeal [71] and perivascular [72,73] origin populate the lesion cavity, where they interact with reactive astrocytes to oppose the glia limitans and form a secondary fibroblast cell layer with the deposition of ECM to form a basal lamina [74,75]. Once in the lesion cavity, infiltrating cells secrete numerous extracellular matrix molecules, including collagen –I associated with the maturing fibroglial scar [76]. In vitro assays have shown that fibroblasts are inhibitory to axonal growth and axons grown in co-culture will avoid growing on their surfaces [75,77]. Similar behavior has been noted in vivo [78,79] and in the rare instance that axons traverse the glial scar their growth is usually attenuated at the fibroglial interface and associated basal lamina [78,79]. More recently, it has been demonstrated that macrophages are responsible for fibroblast recruitment to the injury site and depletion of hematogenous macrophages results in reduced fibroblast density and basal lamina formation in the lesion site, and this is associated with increased axonal growth [80]. Furthermore, the proliferation of fibroblasts can induce fibrosis around biomaterial implants [81,82,83] a particular caveat of biomaterial use [84], and have been implicated in the relatively poor implant–host integration in some implanted scaffolds [65,85].

### 3.2. Macrophage Recruitment after CNS Injury

In contrast to PNI where all macrophages rise from local or hematogenous origin, two divergent yet similar populations of macrophages play a central role in all forms of physical CNS injury. The first are those that are tissue resident macrophages in the CNS, including microglia dispersed throughout the parenchyma and in border regions such as meningeal, perivascular and choroid plexus macrophages. Elegant fate-mapping has determined that collectively these CNS resident macrophages arise from yolk sac precursors during embryonic development and remain as a stable population with self-renewal capacity upon injury, inflammation, neurodegeneration and repair [86,87]. The second important population are monocyte-derived macrophages which are recruited upon specific signals to infiltrate the CNS, contributing to localized inflammation and mechanisms of repair [87]. Although indistinguishable by standard techniques, with the evolution of more sophisticated technology, non-redundant roles for these macrophage populations are beginning to emerge.

Following an acute traumatic insult in models such as SCI, micro-stab or laser-induced wounds to the CNS, there is an immediate reactive response by microglia followed closely by infiltration of monocyte-derived macrophages [55,56,88]. Within seconds and minutes, microglia react predominantly to the release of ATP from damaged neurons, which changes microglia morphology, enhancing their number and length of processes and chemotactic properties. Macrophages work closely with astrocytes to form the glial scar [89]. In fact, preventing this acute microglial response leads to an increase in the size of the lesion and demonstrates that microglia play an important protective role at this time [88]. G protein coupled purinergic receptors and signaling through the phosphoinositide 3-kinase (PI3K) and Akt signaling pathways, in addition to the presence of NO, are important for driving microglia motility in response to ATP [89,90,91,92,93,94,95,96]. Moreover, ATP has been shown to enhance microglia markers such as Iba1 and C1qR to enhance microglia migration [97,98].

After SCI, infiltrating monocyte-derived macrophages are detected in the spinal cord after 2–3 days, reaching maximum levels by days 7–10 and remain present in the injured cord up to day 42 [99,100,101,102]. They localize around the lesion site, in particular in response to chondroitin sulphate proteoglycan (CSPG) upregulated on the surface of reactive astrocytes [103,104]. Interestingly, CSPG and their covalently bound glycosaminoglycans (GAGs) have important immune-regulatory properties, which can bind L- and P-selectins, and CD44 aids the recruitment and enhanced binding of both microglia/macrophages [103,105,106,107]. In fact, prevention of CSPG formation using xyloside in SCI dramatically reduces the infiltration of monocyte derived macrophages to dramatically impair the repair process [103]. Integrins are also an important driver of this process, where their enhanced expression occurs on blood vessel endothelia, activated microglia and infiltrating macrophages [108]. 

The overriding purpose of both microglia and recruited macrophages in this initial response to injury is phagocytosis. Microglia phagocytose damaged material within the first day of SCI, with infiltrating macrophages following suit. Despite infiltrating macrophages being much more numerous and proficient in this activity, both are responsible for the clean-up of damaged tissue, apoptotic cells, myelin and red blood cells resulting from hemorrhage, all of which play a fundamental role in preparing the injury for repair [102,109]. At this point, macrophages concurrently adopt an inflammatory phenotype in response to CNS injury which is essential for clearing any bacterial infections, sterilizing the site of injury and stimulating the recruitment of more immune cells. It is at this tipping point that an over-riding inflammatory burden largely mediated by microglia/macrophages and the presence of a glial scar are prohibitive to the repair process in the CNS [56,63,110,111].

### 3.3. Macrophage Polarisation and Roles during CNS Injury

**Figure 3:**
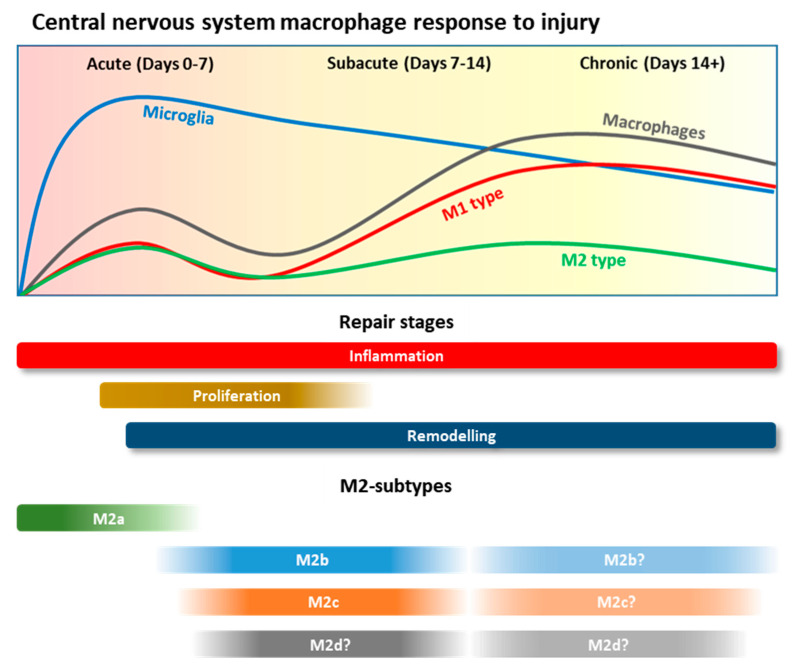
Temporal changes in macrophage phenotype during CNS injury and repair. Timelines for cell infiltration, repair stages and M2 phenotype transition in the wound site were obtained from the following sources.

Upon initiation of an acute traumatic injury, overall phenotypic analysis indicates that microglia and macrophages adopt an initial inflammatory ‘M1’ state [112] (Figure 3). This is defined by enhanced expression of iNos, GM-CSF, CCL2, CCL3, Cox2, the release of pro-inflammatory cytokines TNF, IL-1β and IL-6, and metalloproteinases such as MMP-13 [110,111,112,113,114,115,116]. Microglia rapidly secrete NO and TNF where expression peaks early at 1 h post injury, whereas IL-1β expression also occurs early within 1–12 h, with infiltrating macrophages contributing to the same pool of inflammatory mediators upon recruitment [101,115,117,118].

During the reparative process of central nerve repair, macrophages adopt an M2 phenotype, which can be further annotated into an M2a, M2b and M2c profile [48]. It is believed that as a wound progresses, M2a macrophages are present in the initial phases of injury (days 1–4) followed an M2b phase which occurs during the proliferative phase of wound repair (day 4–7), followed by M2c in the remodeling phase of repair (day 4 onwards) [48]. Many of our insights are gleaned from in vitro assays and there is a real urgency to better define and characterize these M2 sub-types in real-time during SCI [48]. For now, we know that collectively they are typically characterized by high levels of Arginase 1, Ym1, and CD206 and the secretion of IL-10 and TGFβ [100,112]. Upon SCI, M2 co-exist with M1 macrophages, but are present in low numbers, yet they proportionally increase and peak between day 4 and 7 post-injury and even later at day 10 [110,114,119]. It is unclear whether M2 macrophages originate from monocytes or microglia, but it has been postulated that they are converted from an M1 population [117,120]. M2 macrophages exhibit tissue repair properties, produce anti-inflammatory cytokines and chemokines, increase phagocytic receptors, and upregulate extracellular matrix components and growth factors that aid repair [121,122].

The initial experiments to show that microglia and macrophages in an M2 phenotype play a directly beneficial role in acute CNS injury were undertaken when M2 macrophages stimulated ex vivo with nerve fragments were implanted into the parenchyma of rodents, with SCI resulting in tissue repair, partial recovery and enhanced locomotion [6,118]. The neuroprotective capacity of autologous administration of M2 macrophages has been confirmed in bone marrow chimeric models, in addition to human pre-clinical trials and other models of CNS damage such as retinal injury [119,123,124,125,126]. M2 macrophages play a critical role in dampening the inflammatory response, where IL-10 expression is essential for limiting IL-6, TNF and IL-1β secreted by M1 macrophages [127]. Moreover, M2 macrophages produce metalloproteinases such as MMP-13 and MMP-9, which have been shown to contribute to the degradation of CSPG [104]. They also secrete neurotrophins such as insulin-like growth factor (IGF-1), brain derived neurotrophic factor (BDNF), nerve growth factor (NGF) and oncomodulation [128,129]. Importantly, they also contribute to the proliferation and differentiation of neural progenitor cells [130,131] and, similarly, NPCs have been shown to skew inflammatory macrophages towards an M2 phenotype. In addition, oligodendrocyte precursor cell differentiation and maturation, critical for the demyelination of regenerating axons, can be facilitated through activation of M2a and M2c macrophages [132] and in the injured CNS M2b macrophages may reduce axonal dieback [133]. In vitro, M2 macrophage-stimulated astrocytes can inhibit the proliferation of both M1 and M2 macrophages and decrease the production of pro-inflammatory factors [134].

## 4. Macrophage Response to Current Peripheral Nerve Repair Strategies

PNI is a common neurological damage that affects individuals from all walks of life and, in many of them, it exhibits an incomplete recovery that results in disabilities, personal distress and societal costs [135]. It is estimated that approximately twenty million people in the US suffer from PNI and there are over 300,000 cases per year in Europe. Its causes include trauma, diseases or the outcome of surgical procedures, and can result in permanent muscle impairment and altered sensation in the affected area [136].

### 4.1. Auto and Allografts

Patient autografts are the most common approach to repair long distance (>3 cm) peripheral nerve defects. Grafted nerves consisting of a single piece, as a cable (where several parts of a nerve graft are gathered together by sutures or glue), a trunk (where a segment of a nerve is used to approach the two stumps of the injured nerve), interfascicular (where strands of grafted nerve between groups of fascicles), or vascularized (a nerve graft which maintains its blood supply through its vascular pedicle that is micro-surgically anastomosed to the recipient site vessels) [137,138,139]. To date, the sural, lateral antebrachial cutaneous nerves, the anterior division of the medial antebrachial cutaneous nerve, the dorsal cutaneous section of the ulnar nerve and the superficial sensory section of the radial nerve have all been used as donor autograft tissue [140]. 

However, although patient derived, even these procedures can stimulate an innate immune response. Roballo et al. demonstrated that high levels of macrophage and B-cell infiltration were evident in autografted nerves at day 3 and day 7 post-op. Interestingly, macrophage and B-cell levels were also found to be significantly higher than the corresponding response in allografted tissue without immuno-suppression [141]. Although it might be assumed that allografted tissue, especially in the absence of immune suppression, would trigger a higher rate of macrophage infiltration than autografts, the authors highlighted several possibilities for this phenomenon; specifically, that this more gradual immune response to the allografted tissue was due to the epineurium present around peripheral nerves, which may slow immune cell infiltration. T reg cells were not found to predominate and it was also suggested that myelinating allograft Schwann cells may dampen the cytotoxic response. A similar, more rapid macrophage response in autografted peripheral nerves when compared to grafts made from acellular muscles and veins was also previously described by Fansa et al. [142]. 

#### 4.1.1. Decellularized Nerve Grafts

Decellularized nerve grafts (DNGs) have become the most recent approach to peripheral nerve repair to achieve clinical approval; for example, the Avance^®^ Nerve graft by AxoGen. These grafts are human cadaveric nerves that have been processed to remove immunogenic material such as cellular DNA while retaining critical, pro-regenerative topographical and extracellular matrix-based signals [143]. Thus, they represent a particularly attractive biomaterial approach as they do not require long-term immunosuppression and retain key regenerative cues in terms of both nerve micro-architecture and extracellular matrix [144]. Clinical reports on use of these DNGs have been generally positive. Implanted grafts have been reported to be well tolerated and minimally immunogenic across a number of case reports [145,146,147,148].

However, the use of DNGs have identified that their ability to repair over longer distances was directly related to immune response. Specifically, Pan et al. recently demonstrated that significantly reduced axonal repair and angiogenesis were present in 4 cm DNG implanted rats compared to the use of 2 cm DNGs implanted in rats. Interestingly, there were also found to be significantly higher populations of M2 macrophages remaining in the shorter distance DNG at 4 weeks post-implantation. Distance related effects were also observed to limit the secretion of the pro-repair cytokine IL-4 [149].

#### 4.1.2. Biomaterial Nerve Guidance Conduits

In order to address the donor site morbidity and immuno-suppression issues associated using auto- or allo-grafted tissue, Nerve Guidance Conduits (NGCs) were developed and first clinically approved in 1999 [150]. NGCs function by acting as a bridge between nerve stumps and providing a protective environment for the regenerating axons to grow unimpeded. There are numerous experimental variations of NGCs (reviewed in depth [151,152,153]); however, the majority of clinically approved devices are hollow tubes. Originally, these were fashioned from non-resorbable materials such as silicone or polytetrafluoroethylene (PTFE, Gore-Tex^®^, [154]), however their non-biodegradable nature sometimes necessitated secondary removal surgeries due to fibrotic encapsulation of the implant [152]. More recent and approved NGCs are now based on resorbable biomaterials such as polyglycolic acid (Neurotube^®^, Synovis^®^ Micro Companies, Alliance Inc., Kent, WA, USA), collagen I (NeuraGen^®^, Integra Life Sciences Co., Princeton, NJ, USA) and NeuroMatrix™ (Collagen Matrix Inc., Oakland, NJ, USA) and co-polymers of lactide and caprolactone (Neurolac^®^, Polyganics B.V.) [150].

Since these NGCs have all been approved for use in humans, a high level of safety and efficacy has been reported in each case. Indeed, a recent review by Rbia et al. highlighted the relatively low rate of complications reported for the NeuraGen type 1 collagen nerve conduit (Integra Life Sciences, Plainsboro, NJ, USA) (and the Avance DNG (Axogen Inc., Alachua, FL, USA)) [155]. However, because these implants are designed to be non-retrievable, acceptance criteria are mostly framed in terms of return of function/sensation and patient comfort. It is only possible to assess the macrophage response in cases of adverse events and removal of the implanted NGC in humans. Further studies highlighting the immune response of implanted NGCs can be assessed using pre-clinical animal models. 

**Figure 4:**
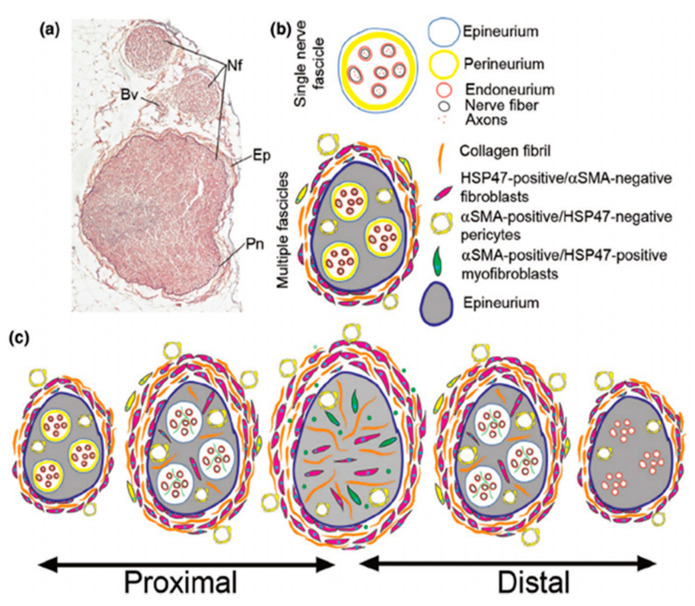
Overview of the scar region of the sciatic nerve for weeks after injury following chronic M1 macrophage activation. The schematic depicts crucial elements of the scar including blood vessels, collagen fibrils and contributing cells in the scar formation process. HSP47—Heat shock protein 47, αSMA—alpha Smooth Muscle Actin (Adapted from [39], John Wiley & Sons, Inc., 2020).

Whether based on a human or pre-clinical model, it is clear that when NGCs do not succeed there is a high probability that failure can be traced back to an uncontrolled innate immune response [156,157]. Typically, a failure to resolve chronic inflammation gives way to the fusion of macrophages to form giant cells, fibroblast recruitment, excessive collagen deposition and fibrous capsule formation [158]. This foreign body response is particularly relevant in peripheral nerve repair as these macrophage-induced fibrotic capsules may take the form of a neuroma which can result in some discomfort [159,160]. In a recent study by Fertala et al., using a nerve guidance conduit derived from porcine submucosa extra-cellular matrix, the Axoguard Nerve Connector (Axogen Corp), it was found that a fibrotic response was evident internally and externally to the conduit [39]. Furthermore, Fertala et al. demonstrated that the constituent parts of the fibrotic bodies were spatially dependent (Figure 4). In some cases, this fibrotic encapsulation is due to an overly-quick degradation of the NGC; for example, the thin-walled NEUROLAC^®^ (PolyGanics) NGCs [161]. However, the use of poly(L-lactide) and poly(ε-caprolactone) NGCs can also successfully protect against macrophage infiltration and scar tissue formation in peripheral neurolysis models [162].

## 5. Biomaterial Approaches to Direct Macrophage Phenotype in Peripheral Nervous System Repair

Recently, a concentrated effort has been made to integrate stimuli into biomaterial NGCs that can regulate the behavior, adhesion, morphology, phenotype and other biological characteristic of macrophages in peripheral nerve repair.

### 5.1. Biomaterial Composition

#### 5.1.1. Organic

Biopolymers such as collagen, chitosan, fibrin, keratin and laminin have been investigated for their ability to enhance peripheral nerve repair and their potential immunomodulatory effect. Specifically, due to their natural origin, these materials have the capacity to mimic the extracellular environment and, in addition, they possess bioactive molecules that, when released, can modulate cellular behavior. Stenberg et al. found that chitosan nerve guides can trigger a pro-regenerative modulation by promoting the differentiation of macrophages into the M2 phenotype. Specifically, that macrophages seeded in vitro on chitosan films were characterized by high levels of markers CD163 and CD206 [163]. Similarly, keratin can have a beneficial role during inflammation, because it was demonstrated that it has the capacity to upregulate IL-10 mRNA relative expression in macrophages after LPS stimulation in vitro [164]. Furthermore, a recent study revealed that the sustained release of collagen VI within a polycaprolactone (PCL) electrospun conduit was beneficial for nerve regeneration by promoting M2 macrophage polarization [165]. Keratin, for example, possesses the peptide-binding motif leucine-aspartic acid-valine, which is recognized by the α4β1 integrin. Because this integrin is also present in macrophages, it is suggested that this ligand–receptor relationship can affect macrophages attachment and behavior [166]. Collagen can bind directly to immune cells expressing various receptors, such as integrins, discoidin domain receptors DDR1 and DDR2 and leukocyte-associated immunoglobulin-like receptor-1 (LAIR-1) [167]. This latter, in particular, has high affinity to collagen I, II, III and XVII [167] and it was demonstrated that its interaction with the collagen III peptide ligand can inhibit inflammation by reducing IFN-α secretion from M1 macrophages [168]. Moreover, collagen VI was found to act as a chemoattractant for macrophages and it can modulate macrophages’ phenotype through protein kinase A (PKA) and protein kinase B (AKT) signaling pathways [27]. Fibrin and its precursor fibrinogen, by comparison, are thought to interact with Toll-like receptor 4 (TLR-4) and integrins αmβ2, αxβ2, Mac-1 and CR-3; however, they also demonstrated to have opposite effects on macrophages’ polarization [169]. In particular, macrophages seeded on fibrin gel were found to secrete predominantly anti-inflammatory cytokines, whereas fibrinogen induces macrophages towards a pro-inflammatory state [169].

#### 5.1.2. Synthetic Polymers

Another source for the fabrication of biocompatible NGCs comprises the use of synthetic polymers. The most common synthetics-based materials used in research are the polyesters. These polymers have a controllable degradation rate and have increased mechanical properties if compared against its organic counterparts [170]. However, to date, few research groups have studied peripheral nerve macrophage activity in these types of guidance constructs. For instance, Jia et al. in 2018, developed an electrospun poly(L-lactic acid-co-ε-caprolactone) (P(LLA-CL)) NGC, testing it in sciatic nerve injured Sprague Dawley rats. Through histological analysis, it was observed that in this injury model macrophages were localized between the infiltrated Schwann cell population and conduit space [171]. A year later, Sarhane et al. showed in an in vivo experiment, conducted on adult male Sprague Dawley rats, that macroporous nanofiber wraps made of nonwoven elctrospun PCL fibers, had a lower inflammatory response in comparison with conventional epineural repair. This is due to the lower number of M1 macrophages at the repair site. In addition, it was assessed that, in the NGCs, the proportion of type M2 macrophages was much higher than in the control groups [172]. In 2020, Zhang et al., used poly(d,l-lactide-co-caprolactone) (PLCL) films coated with graphene oxide (GO) that could promote the migration of Schwann cells with the presence of M2 macrophages [173]. In addition to these findings, the three studies showed that by physically or chemically modifying the architecture of the NGC, macrophages could be polarized into a M1 or M2 phenotypes. These physical-chemical modifications of NGCs are discussed in the following section. 

### 5.2. Physical or Chemically Modified Biomaterials

#### 5.2.1. Physical Modifications

Physical modification of nerve grafts has been widely studied and they are well known to influence cell behavior and, consequently, the regeneration of peripheral nerves. Traditionally, techniques focused on extrusion, injection molding, centrifugal casting and freeze-drying. However, many advanced fabrication techniques are currently being explored to obtain specific characteristics at the nanoscale, regarding their surface topography and fiber alignment [174]. 

It is hypothesized that macrophage polarization is susceptible to surface topography and configuration because the shape of macrophages themselves could affect the phenotype [175]. (Table 1). Typically, the M2 phenotype is usually characterized by an elongated shape, whereas M1 macrophages tend to have a more rounded shape [175,176]. As a demonstration that affecting cell conformation can have a direct effect on their function, an in vitro study conducted by McWorther et al. in 2013 showed that, using a micropatterning approach, macrophage elongation can lead to a higher expression of arginase-1, CD206 and YM-1 markers, therefore, promoting their polarization towards a pro-repair M2 phenotype [176]. These results agreed with the study performed by Jia et al., wherein NGCs made of electrospun P(LLA-CL) nanofibers were used to compare the effects of aligned and random fibers on macrophages’ polarization. The experiments, which were performed both in vitro and in vivo on rats, demonstrated that aligned nanofibers can promote polarization of macrophages towards the anti-inflammatory M2 phenotype, increasing Arg1, IL-10 in vitro and a higher M2/M1 ratio in vivo. In contrast, randomly oriented nanofibers showed opposite results. Furthermore, macrophage elongation was more significant in aligned nanofibers [177].

Other physical approaches involve surface modification by creating micropatterns; for example, the creation of microgrooves on the surface of the biomaterial [171]. The effects of microgrooves are evident in an in vitro study in which different micropatterns designed on perfluoropolyether (PFPE) surfaces resulted in significant changes in macrophage behavior. Here, it was observed that macrophages cultured on post patterns were more likely to express an anti-inflammatory response than line patterns and greater distances between posts were associated with a stronger anti-inflammatory behavior [178]. The use of topographical cues was also analyzed in the study performed by Zhang et al., where a PLCL NGC was micropatterned with ridges and grooves and further modified with the application of GO nanosheets. The microgrooves together with the GO modification induced a change in macrophages shape and their presence was associated with high levels of anti-inflammatory biomarkers, such as arginase 1, IL-10 and Sirtuin 1(SIRT1). Furthermore, by analyzing the contribution of the two individual modifications, it was demonstrated that the GO layer had a stronger effect on macrophage phenotype than the micropattern approach. It was found that the anti-inflammatory markers (arg1, IL-10, SIRT1) were expressed in high levels using the flat-GO film, compared to the micropatterned-only [173].

An emerging area of interest for the modulation of macrophage behavior by physical cues is the control of substrate stiffness [175]. Recent research has found stiffness of the substrate directs macrophage morphology, with soft substrates resulting in a rounded morphology and a more spread shape on stiffer substrates. In tandem, harder substrates resulted in increased TNF-α, IL-1β and IL-6 secretion, and a more pronounced foreign body response [179]. Similarly, Sridharan et al. demonstrated that collagen-based substrates could be specifically fabricated to induce a more favorable surface stiffness and in vitro macrophage response [180]. Although the effects of stiffness on macrophages is currently the subject of a large volume of research, there is less information available on the effect of peripheral nerve resident macrophages. However, recent work has found stiffness is of high importance for the correct fabrication of biomaterials for peripheral nerve repair. Stiffness gradients have been found to elicit significant changes in both neuronal [181,182] and Schwann cell behavior [183,184,185].

Similar to the previous study by Zhang et al. [173], it is important to highlight the complex interplay between the biomaterial composition, its alignment and stiffness. For example, Friedmann et al. demonstrated that macrophage polarization exhibited a wound healing phenotype in stiffer matrices via expression of relevant cytokines (IL10, IL12 and TNFα). The presence of sulfated and non-sulfated GAGs in the collagen matrix was found to inhibit macrophage polarization [186]. 

Lastly, fiber and pore sizes of a conduit are another parameter to consider to elicit a specific macrophages response. In their study using PCL NGCs, Sarhane et al. suggest that a specific fiber and pore size, respectively 1.1 ± 0.5 µm and 6 ± 2 µm after heat-treatment, enables trapping of macrophages and affects their polarization into the M2 phenotype, thereby providing an adequate microenvironment for peripheral nerve regeneration. [172].

#### 5.2.2. Chemical Modifications

In addition to the physical modifications that can be performed on a nerve conduit, the surface chemistry of a material plays a central role in eliciting a beneficial inflammatory response (Table 2) [187]. In this context, the crosslinking procedure applied to the nerve conduit can be critically relevant because it strongly affects its mechanical properties and stiffness and, consequently, the macrophage response [180]. Some of the chemical crosslinking agents commonly used are formaldehyde, hexamethylene diisocyanate, glutaraldehyde (GA), polyepoxy compounds, carbodiimides (EDAC, EDC) and genipin [180,188,189]. The effects induced by the crosslinking procedure on macrophages phenotype are described in a 2019 study by Kočí et al. Here, they compared the effects of genipin and formaldehyde on collagen-based nerve guidance conduits. Interestingly, the authors determined that genipin crosslinking was associated with an increased level of IL-10 and a reduction in TNF-α. Conversely, these were more expressed in the conduits crosslinked with formaldehyde. Therefore, the results suggested that genipin crosslinking can be a valuable method to direct macrophages polarization towards an anti-inflammatory phenotype [188].

In addition to chemically crosslinking the biomaterial, creating a biomaterial for electrochemical stimulation can also modulate macrophage behavior in the peripheral nerve. Graphene oxide (GO) has recently gained interest due to its bio-adaptability and electroconductivity, which are necessary to facilitate the transmission of action potentials [192,193]. Several relevant studies already demonstrated that the chemical modifications associated with GO enable it to regulate the inflammatory response by affecting macrophages’ behavior [173,194]. In addition, other experiments focused on the use of GO-modified conduits together with an external electric current. Dong et al. demonstrated that the association of graphene-based conductive fibrous scaffold and exogenous electrical stimulation is an efficient method to improve nerve regeneration and direct macrophage polarization. Specifically, it was assessed that the graphene-based conduits, when crossed by an external current, had a significantly increased number of macrophages positive for CD163, a marker of the anti-inflammatory phenotype, in comparison to the other scaffolds analyzed [189]. In a similar study by Agarwal et al., a graphene crosslinked collagen-based nerve conduit, when seeded with raw 264.7 macrophages in vitro, induced a high expression of anti-inflammatory markers CD163 and CD206. Consequently, their results suggest that the presence of graphene and collagen within the scaffold could stimulate macrophages polarization towards an anti-inflammatory state [190]. 

Nanodiamonds (NDs) represent a non-graphene carbon-based approach to chemically manipulating macrophage behaviours in PNI. Contrarily to graphene, NDs are not electrically conductive, but still have an excellent biocompatibility [191]. In addition, a study conducted by Quian et al. highlighted their ability to modulate the immune response. Using microporous ND/PCL nerve bridges, they showed that ND constructs, in comparison to PCL only, were associated with lower levels of pro-inflammatory markers and higher levels of anti-inflammatory markers [191].

### 5.3. Therapeutic Molecule Incorporation

#### 5.3.1. Small Molecule Drugs

Therapeutic treatment with other biological compounds revealed another promising approach for PNI inflammation (Table 3). Miconazole, which is an antifungal medication, was found to exert an anti-inflammatory effect by suppressing the NF-kB signaling pathway, thus promoting M2 macrophage polarization [195]. Thrombomodulin, a transmembrane glycoprotein, was revealed to have the capacity to enhance nerve regeneration and to induce M2 macrophages’ polarization by activating the STAT6-PPARγ pathway [196]. Lastly, treatment with vitamin B complexes, namely vitamins B1, B2, B3, B5, B6 and B12, was found to be beneficial against inflammation, because it was demonstrated that its use can accelerate the transition from M1 to M2 macrophages and it can reduce the expression of pro-inflammatory cytokines while increasing that of the anti-inflammatory [197].

#### 5.3.2. Proteins/Macromolecules (Cytokines, Peptides, Antibodies)

An additional approach can be the use of biological factors such as cytokines. For this reason, several studies proposed their use to regulate macrophages polarization after PNI. Mokarram et al. found that the local delivery of IFN-γ or IL-4 within a polymeric guidance channel is effective in inducing macrophage polarization towards the M1 phenotype and M2 phenotype, respectively. In this case, the delivery approach was performed by filling a polysulfone tube with agarose hydrogel mixed with the cytokines [198]. Similarly, the addition of the chemokine fractalkine within agarose to a polysulfone-based scaffold demonstrated promising results. Fractalkine was shown to control monocyte subtype recruitment, by promoting the infiltration of fewer macrophages with an anti-inflammatory phenotype, to benefit axonal growth and the electrophysiological outcomes [199]. A study conducted by Potas et al., by comparison, showed that IL-10 functionalized PCL-based scaffolds, conjugated to the surface of the fibers, can polarize macrophages to the M2 phenotype in vivo [200]. 

#### 5.3.3. Cellular Therapeutics

Another encouraging biological method is the delivery of cells to the site of injury. Delivered stem cells particularly have the capacity to regulate the inflammatory response. This has been explored by Li et al., whereby they demonstrated that epidermal neural crest stem cells (EPI-NCSCs), seeded on ECM/PLGA constructs, were able to regulate the inflammatory microenvironment by increasing the levels of anti-inflammatory cytokines and decreasing those of pro-inflammatory cytokines [201]. In addition, skin-derived precursor Schwann cells (SKPSC) showed immunomodulatory properties. In particular, it is suggested that these cells can promote the pro-healing macrophages’ phenotype, since they were found to have the capacity to increase the gene expression of arginase 1 in macrophages in vitro and the number of CD206^+^ cells in vivo. However, given that SKPSCs also express high levels of IL-6, their use in combination with anti-IL-6 treatment can be beneficial to avoid detrimental effects associated to this pro-inflammatory cytokine [202].

#### 5.3.4. Gene Therapy

Among the paracrine factors secreted by mesenchymal stem cells, exosomes are considered one of the most important immunomodulatory mediators. Therefore, they could be promising candidates for the regulation of the inflammatory microenvironment following PNI [203]. Exosomes are known to be carriers for miRNAs which have been shown to have an important role in cell-to-cell communication and peripheral nerve regeneration [204]. The role of exosomes in cell-to-cell communication was highlighted by Simeoli et al., who showed that, after spared nerve injury (SNI) in mice, DRG neuron cell bodies can release exosomes containing miRNAs, such as miR-21-5p. These can be phagocytosed by macrophages, thus affecting their phenotype. In particular, they found that the upregulation of miR-21-5p expression enhances the M1 phenotype and, furthermore, the use of miR-21 antagomir or the conditional deletion of miR-21 in sensory neurons can modulate the inflammatory response by reducing the number of M1 macrophages and increasing that of M2 macrophages [205]. 

Similarly, a study found that microRNA-23a expression is upregulated in DRG neurons after SNI and its subsequent expression in macrophages could promote their polarization towards the pro-inflammatory phenotype by inhibiting A20 and activating the NF-kB signaling. In addition, they demonstrated that the local delivery of miR-23a antagomir via extracellular vesicles (EVs) reduced M1 macrophages and increased M2 macrophages in vivo in mice models [206]. Lastly, miR-223 has an important role in macrophages’ polarization since it was found that M2 macrophages and M2 macrophage-derived micro-vesicles (MVs) are characterized by a significantly higher miR-223 expression compared to the pro-inflammatory phenotype. This miRNA was also found to be particularly beneficial for peripheral nerve regeneration, because its downregulation was revealed to inhibit Schwann cell migration and proliferation and to reduce the production of laminin and NGF [207]. In conclusion, these studies highlight the central role of miRNAs as modulative agents during inflammation and they suggest promising approaches to control macrophages’ phenotype by affecting their miR expression. This, in turn, could result in novel therapeutic treatments to enhance peripheral nerve regeneration.

**Table 3:** Therapeutic molecule incorporation to induce macrophage polarization during PNI.

TECHNIQUE/PRODUCT			
Biological Approach	In vivo	Immune Response	Ref
**PCL/collagen VI nerve conduit**			
Sustained release of Collagen VI	Sciatic nerve in SD rats	Promotes polarization of macrophages towards M2 phenotype	[165]
**Polysulfone nerve guidance channel**			
Local delivery of IL-4 or IFN-γ	* Sciatic nerve in adult Lewis male rats	IL-4 or IFN-γ polarizes macrophages towards M2 and M1 phenotypes, respectively	[198]
**PCL nanofibrous scaffold**			
Local delivery of IL-10	Sciatic nerve in Wistar rats	IL-10-conjugated PCL scaffold induces M2 polarization	[200]
**Polysulfone nerve guidance scaffold**			
Local delivery of Fractalkine	Sciatic nerve in Lewis rats	Fractalkine promotes the recruitment of anti-inflammatory macrophages	[199]
**Therapeutical treatment**			
Miconazole	* C57BL/6 mice	Suppresses M1 phenotype and induces M2 phenotype	[195]
Thrombomodulin	In vivo: sciatic nerve in male SD rats and In vitro: THP-1 cells	Promotes nerve repair by M2 polarization	[196]
Vitamin B	Femoral nerve in AO rats	promotes M2 polarization, reduces pro-inflammatory cytokines and increases anti-inflammatory cytokines	[197]
**ECM/PLGA bridge**			
EPI-NCSCs delivery	Sciatic nerve in adult female SD rats	Delivery decreases inflammatory fibroblasts and increases the M2/M1 macrophages ratio	[201]
**Cell Transplant**			
SKPSCs delivery	In vivo: Sciatic nerve in adult Lewis rats; In vitro: unprimed adult macrophages with SKPSC-conditioned medium	SKPSCs enhances expression of arg1 and an increases number of CD206^+^ macrophages. Nonetheless, these cells were associated with high levels of pro-inflammatory cytokines	[202]
**Gene therapy—miRNA**			
miR-21 antagomir or miR-21 deletion	In vivo: spared nerve injury in mice; In vitro: PECs with exosome-enriched media from sensory neurons	miR-21-5p expression was associated with pro-inflammatory phenotype. Inhibition of miR-21-5p promoted M2 phenotype	[205]
EV-miR-23a	antagomir In vivo: spared nerve injury in mice; In vitro: coculture of DRG neurons with mouse macrophages	EV encapsulated miR-23a promoted M1 polarization. Inhibition of miR-23a reduced M1 macrophages numbers and increases M2 macrophages	[206]

Annotation: Sprague Dawley^®^ (SD); interleukin-10 (IL-10); Albino Oxford (AO); epidermal neural crest stems cells (EPI-NCSCs); Arginase-1 (arg1); Peritoneal exudate cells (PECs); Extracellular vesicles (EVs); * In vitro and in vivo.

## 6. Biomaterial Approaches to Direct Macrophage Phenotype in CNS Repair

The use of biomaterials for CNS repair, particularly for SCI research, have flourished over the past 30 years and encompass two broad categories—guidance scaffolds containing architectures similar to those of peripheral nerve scaffolds [208,209,210] and injectable formulations that can more easily fill smaller irregular cavities or lesions deeper in the CNS [211,212,213]. 

The salient feature of any biomaterial implant is to act as a trophic environment for host cell infiltration, colonization and differentiation but, more importantly for CNS repair strategies, it must serve as an attractive trophic environment for regrowing axons [214,215]. However, biomaterials implanted into the lesion cavity during the acute or chronic stages of injury encounter an environment harboring a cycling M1 macrophage population and the lesion site maintained in a chronically inflamed state. 

In addition, for any biomaterial implant there is the added caveat of the physical act of scaffold insertion which may induce further inflammation and induce a foreign body response [66], particularly if there is hemorrhage. Long known as a caveat of electrode implantation in the CNS, and essentially a stab injury, it can induce a strong reactive glial response along the track of the implant to form a glial scar [216] that is exacerbated by fibroblast and macrophage influx [84,217]. More recently, it has been shown that hydrogel biomaterial implantation can induce a multicellular foreign body response in the CNS tissue that mimics conserved elements of CNS wound healing [66]. Therefore, it is highly desirable for biomaterial implants to be able to control macrophage induced inflammatory responses. 

Mounting evidence indicates that biomaterial composition can have a strong effect on immune response [156]. Strategies employed to enhance the bio-functionality of injectable- and scaffold-hydrogels can be improved by the addition of extracellular matrices (ECM), ligands, proteins and cells to encourage axonal and cellular infiltration [218,219,220] and synergistically they can also influence the immune response in the lesion cavity [221,222]. Furthermore, incorporation of explants, biological, chemical physical, chemical parameters numerous studies have demonstrated the ability of biomaterials to influence macrophage behavior and encourage phenotypes necessary for tissue remodeling and healing [48].

### 6.1. Macrophage-Based Clinical Trials in the CNS

There are currently no clinically approved CNS repair strategies and limited progression of macrophage therapeutics to clinical trials for CNS injuries. A 2005 clinical trial used autologous macrophages to promote axonal regrowth in SCI. The method consisted of injecting macrophages directly into the injured site 14 days post-injury. Although three of the eight patients participating in this study recovered neurological and motor functions, unfortunately two patients presented pulmonary embolism and one patient had a case of osteomyelitis. This trial has not received any updates and has remained suspended since 2009 [119]. A 2013 study investigated levels of proinflammatory cytokines in blood after SCI. During the first 3 days post-injury, elevated levels of IL-6, IL-9, IL-16, CCL4 were observed. After 7 days post-injury, however, the only cytokine that presented a reduction was IL-9. They concluded that further clinical studies were necessary to fully understand the role and mediation of proinflammatory cytokines after an acute SCI [223]. 

### 6.2. Peripheral Nerve-Based Implants in the Lesioned Cord 

Unlike lesions to the peripheral nervous system where autografts and allografts can be used to repair damaged and transected nerves, strategies employing replacement adult CNS tissue are not possible for the injured spinal cord. However, because of their trophic ability to support axonal growth, implanting peripheral nerve bridges to circumvent the inhibitory lesion site was undertaken in some of the first experiments conducted using non-CNS material over 40 years ago [224,225,226]. By utilizing the longitudinal alignment of axon tracts in peripheral nerves, injured descending brain stem [224] and propriospinal cord neurons [226] grew through the grafts and bypassed the inflamed cord lesion site [224]. Implanting PN tissue directly into the lesion core has the potential to influence many more ascending and descending axons [227], and multiple studies demonstrate the ability of grafts to host regrowing axons [228]. However, allografts can require immunosuppression to avoid adverse immune responses and further expansion of the lesion core [229]. These findings have led to the development of decellularized grafts that eliminate the need to harvest PN tissue from the patient, while also suppressing potential host-graft immune responses [230] and supporting axon growth [231].

### 6.3. Biomaterial Composition

#### 6.3.1. Organic

Collagen, due to its ubiquitous distribution in the ECM of many body tissues, its ease of use [232], biocompatibility, capability to support cell growth, and biodegradability has been extensively used to produce hydrogel scaffolds for implantation into lesion sites in the brain [233,234,235] and spinal cord [236,237,238,239]. When combined with rat mesenchymal stem cells, collagen scaffolds promoted significant recovery of nerve function in a rat model of SCI and were accompanied by reduced apoptosis and glial fibrillary acidic protein, less macrophage infiltration and greater macrophage M2 polarization [240]. However, collagen type I is also highly expressed in the lesioned cord during the scar-forming phase and induced astrocytic scar formation via the integrin–N-cadherin pathway [65]. Because collagen I is primarily produced by invading fibroblasts [76] sensitive to M1 macrophage signaling [80] and coupled with astrocyte-macrophage reciprocal inflammatory signaling [70], this may indicate a potential caveat for the use of collagen based materials in lesioned CNS tissue. Nonetheless, hybrid collagen scaffolds containing porcine decellularized ECM mixed in a respective 3:1 ratio with collagen reduced the number of ED-1+, CD86+ (M1) cells and increased the number of ED1+, Arginase-1+ (M2) cells, along with an increased expression of molecules associated with an M2 (CD206, arginase1, and IL-10) phenotype in the injured spinal cord [241].

Furthermore, using the collagen derivative gelatin as a coating of implant-induced stab lesions in the brain reduced the inflammatory response relative to uncoated needles and the numbers of ED1-positive cells in the lesion track [242]. Similar reductions in CD68-positive macrophages/microglia were noted after implantation of gelatin sponges containing bone marrow mesenchymal stem cells into the injured rat spinal cord [243]. Furthermore, incorporation of the CSF1R inhibitor into gelatin scaffolds inserted into the injured rat spinal cord significantly reduced CD68-positive reactive microglia/macrophages and mRNA levels of pro-inflammatory factors [244].

Hyaluronic acid (HA), is the anionic non-sulphated glycosaminoglycan found ubiquitously throughout the CNS, particularly around perineuronal nets [245]. Evidence suggests that HA alone is immunomodulatory and, with nanofibers, capable of significantly reducing LPS-induced M1 gene (iNOS) expression in macrophages and induced release of M2 cytokines (M-CSF, IL-10) in vitro [246]. HA is not structurally inert and the naturally occurring high molecular weight form can be broken down into lower molecular weight fragments [247]. Fragment size can induce differential responses in macrophages, with low molecular weight HA polymers upregulating pro-inflammatory genes, enhanced TNF-α and nitric oxide secretion [248]. High molecular weight HA (mwHA), in contrast, promotes an alternatively activated-macrophage state, upregulating pro-resolving gene transcription and enhanced arginase activity [248] to generate an M2 macrophage phenotype [249]. Studies where high mwHA hydrogels are implanted into the injured cord reduce the number of ED1+ positive macrophages in the lesion site by over 50% at 1, 3 and 10 days after injury [250], with similar results noted in other studies [251]. Although the mechanism of action is unclear it has been proposed that high mwHA, but not low mwHA, cross-linking with CD44 functions as a novel form of pattern recognition to generate tissue integrity signals that promote the resolution of local immune responses [252]. High mwHA may also play a neuroprotective role and is capable of mitigating LPS induced microglial and macrophage activation [253,254]. Composites of biomaterials with HA such as with PCL spun fibers to produce nanofiber-hydrogels, also have a modulatory effect on macrophages [255]. When implanted into the contused rat spinal cord, greater numbers of M2-type cells were present in the lesion cavity. In addition, M2 but not M1 macrophages appeared to congregate in nanofiber-rich areas within the hydrogel after implantation [255].

Various other extracellular matrix molecules are present in CNS ECM, including fibronectin, collagen IV, chondroitin sulphate proteoglycans (CSPGs), heparin sulphate proteoglycans (HSPGs), laminins and tenascins [256,257]. Some are found ubiquitously throughout the brain and cord (e.g., CSPGs, HSPGs, laminins and tenascins) [256] or densely aggregated as part of the perineuronal nets that surround some neurons [245], whereas others (e.g., collagen-IV) are associated with specific structures such as the basal lamina that surrounds blood vessels and the glial limitans at the periphery [258]. Some types, by virtue of their structure and cellular interactions, are poor candidates for scaffold incorporation. For example, CSPGs are potent axon growth inhibiting molecules [245,259]; however, others such as fibronectin, when aggregated in the CNS, promote features of a classically and alternatively Arginase-1 (M2) activated phenotype in macrophages [260]. Fibronectin mats produced through a mechanically shearing process and implanted into the lesioned rat spinal cord induced prolific ED1-positive macrophage influx within 3 days post-implantation but after 4 weeks when axons had entered the lesion site few remained [261], indicating a cessation of macrophage activity and avoidance of a chronic long-term inflammatory response. Laminin variants, and particularly Laminin-111 [262], provide strong trophic support for growing and re-growing axons [263] and are often incorporated into CNS scaffolds as a whole protein [264] or as ligands containing functional subunits (e.g., IKVAV, LRE) responsible for ECM- and cellular- interactions [262,265]. A variant of laminin, termed poly laminin, can be generated from stable laminin polymers produced in low pH that mimic the shape of polymers produced on cell surfaces [266]. When injected into the lesioned rat spinal cord, poly laminin improved motor function after thoracic compression and reduced the number of ED1-positive macrophages in the lesion site [267].

Several types of linear polysaccharides derived from non-mammalian origin have been used for CNS hydrogel applications [213,218,268,269,270,271]. Alginate hydrogel scaffolds, seeded with bone marrow stromal cells. promoted directed linear axonal regeneration in the injured rat spinal cord [268]. Macrophages migrated into the biomaterial closely opposed to the hydrogel channel walls; however, changes in macrophage numbers or polarization phenotype were not noted [268]. 

Agarose scaffolds generated using freeze drying processes to produce uniaxial channels, containing BDNF and with physical properties matching those of the spinal cord, facilitated cellular and axonal growth, with macrophages observed within the scaffold but with no change in ED-1-labelled cells compared to lesioned controls [269]. However, mixed agarose/polyethylene glycol/carbomer based hydrogels containing human mesenchymal stem cells (hMSCs) increased and/or converted efficaciously M2 macrophages in the injured site [218].

Similar changes in macrophage phenotype were reported in a comprehensive study of scaffolds produced using chitosan and water as a fragmented physical hydrogel suspension (Chitosan-FPHS) [213]. Using in vitro and in vivo testing, the authors demonstrate immunomodulatory behavior of the Chitosan-FPHS—reducing M1 marker protein iNOS by 60% and increasing M2 marker proteins Arg-1 and Ym1/2 by 330% [213]. Chitosan-FPHS scaffolds implanted into the cord after a bilateral dorsal hemisection promoted reconstitution of vasculature, diminished fibrous glial scarring and modulated the inflammatory response; analysis of relative levels of M1 (CD86+), M2 (CD206+, and Arginase-1+), and pan-macrophage/monocyte (CD68+) markers demonstrated a five-fold increase in the M2 macrophage marker compared to lesioned controls [213]. Furthermore, water-soluble chitosan also inhibits the production of pro-inflammatory cytokines in human astrocytoma cells in vitro [270] and prevents oxidative stress-induced amyloid beta formation and cytotoxicity in NT2 neurons [271]. 

#### 6.3.2. Synthetic Polymers

Most synthetic polymer scaffolds used for CNS applications are biodegradable, although not all (e.g., PHPMA and PHEMA scaffolds), and equally encourage axonal growth with various success into the implanted scaffold from the injured neural tissue [272,273,274] with some reporting functional restoration after cord implantation [272,275]. The majority of studies have focused on implantation into the injured spinal cord [273,276,277] although many have also been utilized to treat brain lesions [278,279,280]. Many studies report the presence of macrophages/microglia at the interface between the implanted material and the spinal tissue [275,281,282] or within the implanted material itself [273,274,282]. In addition, a number of studies have reported changes in macrophage numbers [275,283,284] or a shift in the M2:M1 balance [208,273,274] in and around the implants that may be indicative of anti-inflammatory properties of the constituent polymer.

The tunable characteristics of synthetic polymers have been extensively used for CNS injuries and possess several advantages (Table 4). For example, their physicochemical properties are easily modifiable, they form hydrogels easily, their mechanical properties are easily tuned, they can be cast into various shapes, they have ease of control of internal architecture (e.g., pore and channel size) and they have reduced allergenic and immunogenicity risks [232,285]. In addition, polymers can be fabricated as nanoparticles and the combination of their small size, ability to sequester and protect therapeutic cargoes, and improve bioavailability, and controlled release at target sites [286] have attracted considerable interest for delivery to the injured CNS [287].

The uses of methacrylamide (Poly[N-2-(hydroxypropyl) methacrylamide] (PHPMA)) based hydrogels for brain [279] and SCI [275,277] report good integration and axonal regrowth in rodent models. Implantation of scaffolds into the lesion site of the rat cord significantly increased the number of ED-1+ macrophages at the edge of the injury with few penetrating into the matrix 14 weeks after hemisection injury [275], indicating a chronic retention of cells around the implant. Similar findings have been reported with methacrylate (Poly(2-hydroxyethyl methacrylate (PHEMA)) hydrogels which are the most researched non-biodegradable polymer for repair [289], with ED-1+ macrophages reported within the scaffold [290] and ED-1+ /Iba1+ cells surrounding the implanted scaffold [290,291] up to 8 weeks post-injury [291]. Similarly, when PLA and PEAD polymers were injected into the contused rat cord, ED-1+ cells were localized between the scaffold itself and the injured cord tissue 14 days after hemi-section injury [281,292]. 

Other synthetic polymers, however, have been reported to reduce overall macrophage numbers in the lesion site. PLLA multi-channel conduits fabricated with a ladder-like porous channel wall alleviated the infiltration of (Iba1+, CD68+) macrophages/microglia both inside and around the conduits, with the total number reducing much faster over time compared to the untreated control group [283]. A similar reduction in macrophage number was noted in the lesion site after injection of the hydrogel SHIELD, consisting of an eight-armed PEG tethered with proline-rich peptides and a thermoresponsive polymer (PNIPAM) linked to recombinant, engineered proteins composed of seven repeats, 4 weeks after contusion injury in rats [284]. However, in all these studies analysis of macrophage subtypes was not performed to indicate whether the hydrogels can modulate the chronic inflammatory response and if resident cells were polarized to M1 or M2 phenotypes.

Imidazole-poly(organophosphazenes) hydrogels implanted into the lesioned rat cord induced endothelial and fibroglial infiltration, which was accompanied by extensive macrophage infiltration. Four weeks post-injury, many were CD206+, indicating that most possessed an M2 phenotype [272]. Hakim and colleagues (2015), using oligo[Poly(Ethylene Glycol) Fumarate] scaffolds loaded with or without Schwann cells in matrigel ECM and implanted into the completely transected spinal cord, noted no difference in collagen scarring, cyst formation, astrocyte reactivity, myelin debris or the number of CD86+ cells (M1 type) in both polymer groups [208]. However, an increase in CD206+ cells (M2 type) was found in all polymer groups 4 weeks after injury that was only significant in the polymer containing Schwann cells [208]. In a dual study of Hypoxic-ischemic injury in neonatal brain and hemisection injury in an adult spinal cord, poly(glycolic acid)-based scaffolds alone or seeded with neural precursor cells at 8 weeks post-injury exhibited less Iba1+ and CD68+, and more CD206+ staining in the injury epicenter compared to vehicle-injected controls [273]. Furthermore, a similar pattern was noted 8 weeks post-transplantation, indicating a sustained chronic inflammatory response dominated by M2 macrophages [273]. Similar findings were reported by Guest et al. (2018) after spinal cord contusion injury in rats, in which PLGA-poly-L-lysine mixed at a 50:50 ratio was implanted into the contused rat spinal cords [274]. At 12 weeks post implantation when infiltrating cells and axons were established in the degrading scaffold, the expression of three markers of M2 macrophages (CD206+ CD163+ and Arginase-1+) was found in cells in the scaffold, and with few CD74 + cells present, indicating reduced M1 phenotype. Together these studies indicate that PLGA-based polymer scaffolds encourage the accumulation of the M2 macrophages in the implanted material 4+ weeks after injury and may also be indicative of the M2-directed remodeling phase in the injury site [273,274]. 

### 6.4. Physical or Chemically Modified Biomaterials

#### 6.4.1. Physical Modifications

Similar to the findings for peripheral nerve graphs (see Section 5.2.1), modification of the physical macro- and micro-environments of biomaterial implants can have a strong influence on CNS cell behavior. Numerous studies have identified key macro and micro features of scaffolds, such as fiber alignment [293], grooves [176] and nano-patterning [294]. In addition, several studies have highlighted specific responses of different aspects of scaffold design and structure that have particular effects on the macrophage population in the lesioned CNS. One such finding from a number of studies is that biomaterial implantation has a positive effect on the fibroglial scar, reducing astrocyte mediated scaring [295,296] and a concomitant decrease in macrophage number [240,284,295,297]. Although it is difficult to ascertain whether this may be due to the physical filling of the space reducing the ability of macrophages to migrate into and through the lesion site, or is a feature of the implanted material, this effect has been noted in scaffolds generated from synthetic [273,283,284,290] or bio-polymers [240,242,244,250], in addition to synthetically and biologically derived nanoparticles injected directly into the lesion site [297,298,299,300,301]. 

Pore size has been shown to be an important feature for axonal repair in the CNS. Thomas and colleagues (2013) studied this effect after SCI using a variety of PLGA porosities, noting that porosities of 80%, corresponding to channel diameters of 230 µm, contained the largest numbers of regrowing axons [302], indicating that scaffolds with large ‘open path’ channels induce the strongest axonal regrowth [303]. Macrophage polarization also appears to be affected by pore size, with 160 μm pores reportedly encouraging M2 phenotypes [304].

The stiffness of biomaterial scaffolds and hydrogels has a direct influence on macrophage and microglia behavior. In general, stiffnesses that mimic those of the spinal cord and brain (200–600 Pa) [305] induce spherical morphologies, with short lamellipodia and processes, whereas on stiffer substrates (10 KPa) the cells spread more and had longer processes. Furthermore, cells grown on stiffer surfaces were noted to upregulate 15 different pathways related to different inflammatory and pathogenic functions [305]. Similar morphological changes were noted in macrophages by Blakney and colleagues (2012) using a range (130, 240, and 840 KPa) of substrate stiffnesses, and further showed that when challenged with lipopolysaccharide, macrophages increased expression of TNF-α, IL-1β, and IL-6; however, the degree of activation was significantly reduced with the softest hydrogels [179]. In vivo, hyaluronic scaffolds with stiffnesses similar to that of the spinal cord and implanted into the injured rat cord significantly reduced the numbers of ED-1+ cells in the lesion site at 3, 5 and 10 days post-injury compared to controls [250]. Similar findings were also noted by Bakshi and colleagues (2004) using poly(2-hydroxyethyl methacrylate (PHEMA) scaffolds with 85% water content and a compressive modulus of 3 to 4 kPa, inserted into the injured cord with reduced ED-1+ staining around scaffolds 1 week after injury and had disappeared by 4 weeks [306]. 

#### 6.4.2. Chemical Modifications

Biologically derived materials have been extensively used as CNS bridging materials due to their innate biocompatibility and bioactivity, biodegradability, and low antigenicity, and because the exhibit similar soft properties as their target tissues [232]. They are often intrinsically functionalized and can be easily modified to enhance their trophic capabilities. Ranging from whole peripheral nerve explants to more easily engineered single type ECM hydrogels and scaffolds, e.g., collagen and hyaluronic acid, which can be more easily modified to suit lesion type and size, they can also be loaded with various cargoes and cells to boost trophic ability [214,307] (Table 5).

Injectable formulations of acellular peripheral nerve tissue have been developed that form hydrogels once in situ and more efficiently fill the lesion cavity of the injured cord and facilitate cellular transplant efficacy [312], encourage some axonal growth, and also appear to polarize the host macrophage response [211]. Using a unilateral cervical contusion mouse model followed by injection of acellular, enzymatically digested peripheral nerve material 7 days after injury, Cornelison and colleagues (2018) noted a significant decrease in the ratio of CCR7+ M1 to CD206+ M2 macrophage phenotypes in the unilaterally contused spinal cord [211]. 

Injectable extracellular matrix (ECM) derived hydrogels have also been generated from porcine brain [241,313], spinal cord [288] and urinary bladder [288,313] and injected into the lesioned brain [212] and spinal cord [288,313]. Protein analysis revealed the presence of various kinds of cytokines and growth factors, including tissue factor, CD26, endostatin, FGF-1 and -2, IGFBP-2 and -9, and osteopontin in porcine brain decellularized ECM [241]. Gene expression analysis revealed decreased gene expression for M1 and M2 phenotypes two weeks after injury with M1 and M2 macrophages exhibiting spatial differences in the distribution. M2 macrophages mostly accumulated within the hydrogel whereas M1 macrophages remained in the surrounding tissue [288]. Furthermore, the ratio of M2:M1 macrophage subtypes in the injury site may be dependent on ECM hydrogel concentration [212,241] which affects the rheology of the material [175]. The highest M2:M1 ratios were found in hydrogels containing ECM concentrations that produced stiffnesses comparable to uninjured brain [212].

Elsewhere, scaffolds composed of decellularized mammalian ECM have facilitated a constructive host response and diminished the number of M1 macrophages (CD86+/CD68+) around the implant and resulting fibrosis [314]. Furthermore, macrophages exposed to extracellular matrix derived from small intestinal submucosa, urinary bladder matrix, esophageal ECM and colon, in addition to the brain, express a predominant M2-like macrophage phenotype [315]. Although the underlying mechanisms of decellularized tissue ECMs to modulate the innate immune response are not fully understood, enhancing M2:M1 subtype ratios may play a central role [175,316]. This strong immunomodulatory effect is also found in hybrid scaffolds containing porcine decellularized ECM mixed with collagen in a 1:3 ratio [241] which also reduced the number of ED-1+, CD86+ (M1) cells and increased the number of ED1+, Arginase-1+ (M2) cells, along with an increased expression of molecules associated with an M2 (CD206, arginase1, and IL-10) phenotype after cord injury [241].

Similar to the results demonstrated in peripheral nerves, graphene oxide (GO) has also been shown to exert positive effects on macrophage phenotype after surgical insertion into the laterally hemisectioned rat spinal cord [308,309]. ED-1 immunostaining for macrophages 10 days after implantation revealed numerous cells at the interface between scaffold and cord tissue with some infiltration into the rGO scaffold itself that consisted of a mixture M1 (CD80+) and M2 (CD163+) cells [309]. By 30 days after injury, implanted rGO scaffolds exhibited significantly reduced CD86+ M1 macrophages at the cord interface compared to lesion alone, and this was matched by a non-significant trend for increased numbers of CD163+ M2 macrophages [308].

Finally, self-assembling peptides (SAPs) comprise a family of biocompatible, biodegradable and easily modifiable short chain amino acid sequences, [317], capable of forming hydrogels, and have shown considerable promise for CNS injury applications [318]. SAPs have demonstrated good integration into the lesioned spinal cord and promote axonal growth [188,189] and do not induce an immune response with CD68+ macrophage numbers similar to lesioned cord controls as long as 4 weeks after injury [319,320]. Because subtyping into M1 and M2 macrophages was not explored, it is not possible to determine if SAPs may exhibit immunomodulatory functions.

### 6.5. Therapeutic Molecule Incorporation

#### 6.5.1. Small Molecule Drugs

Different organic and synthetic polymers have also been employed as injectable nanoparticle delivery vehicles and are capable of encapsulating, absorbing or conjugating different agents such as steroids (methylprednisolone), antibiotics (minocycline), and chemical (Chicago sky blue) cargoes in a controlled and high reproducible manner [321,322] (Table 6). Nanoparticles generated from natural polymers such as chitosan [299], and synthetic polymers such as polycaprolactone [298] and PLGA [323,324], either alone or complexed with a known or novel therapeutic agent, have all been shown to modulate macrophage responses at the injury site [325]. For example, Park et al. (2019) systemically delivered cargo-free PLGA particles every day for 7 days intravenously following spinal cord hemisection in mice which significantly altered the macrophagic response in the injured cord at 7 and 84 days after injury [324]. Levels of the M1-associated proinflammatory markers iNOS+, CD86+ and MCP-1+ (monocyte chemoattractant protein-1) were significantly downregulated 7 days after injury and were matched by a two-fold increase in CD206+ and IL-10+ M2 macrophages in the injury site and these changes were similarly noted 84 days after injury [324]. 

Acutely administered polycaprolactone nanoparticles loaded with the anti-inflammatory antibiotic minocycline injected directly into the compressed mouse spinal cord were readily taken up by activated microglia and macrophages [298]. M1 macrophage influx peaked at day 3 but was reduced by day 7, whereas M2 macrophages peaked at day 7, indicative of an early transition to pro-reparative stages, and this was mirrored by reduced tissue loss in the wound site [298]. Similar reduced tissue loss was noted using albumin coupled, chitosan stabilized, and cationic PLGA nanoparticles carrying minocycline and methylprednisolone into the contused rat spinal cord [327], although macrophage status was not investigated. Using a similar approach, intraperitoneal administration of minocycline hydrochloride complexed with the polysaccharide dextran sulfate was compared to depot delivered nanoparticles embedded in agarose hydrogel depots placed over the contused rat spinal cord [297]. Both approaches significantly reduced the number of CD68+ macrophages in the injury site 6 weeks after injury but with a greater reduction seen in depot delivered nanoparticles. Subtyping of the macrophages in the injured cord revealed reduced iNOS+/CD68+ cells (M1-type) but did not significantly alter arginase 1+/CD68+ cells (M2-type), suggesting a greater effect of the dextran-minocycline delivery on the M1 phenotype [297]. 

Using larger delivery vehicles to target SCI via the intravenous route was also explored by Saxena and colleagues (2015) and demonstrated the delivery of liposome particles carrying cargoes could be targeted to the injured cord. Liposomes containing the small molecule Chicago sky blue (CSB), a known macrophage migration inhibitory factor (MIF), was delivered 48 h after cord contusion injury [326]. Using qRT-PCR and immunostaining, significant increases in the expression of anti-inflammatory markers arginase I and TGF-β were matched by higher ratios of arginase to CD68+ cells in treated animals, although there was no change in CD68+ cells compared to controls [326]. Louw and colleagues (2016) demonstrated that chitosan polysaccharides designed to deliver miRNA-124 via direct injection into the spinal cord after a lateral hemisection of the rat spinal cord induced a 60% reduction in the number of ED-1+ cells in the injured cord 3 days after injury [299]. Similar reductions in macrophages were also noted after administration of the leech-derived anticoagulant peptide hirudin encapsulated in PLGA microspheres and embedded in the thermo-reversible Pluronic F-127 hydrogels that were injected into the contused mouse spinal cord. Hirudin mediated blocking of thrombin activity reduced the number of CD68+ macrophages relative to controls 30 days post-injury [301]. Interestingly, topical application of the cell cycle inhibitor flavopiridol loaded in PLGA nanoparticles to the lesion site substantially reduced the number of macrophages in the injury site of hemisected rats and was coupled with reduced glial scarring and tissue preservation 6 weeks after injury and improved motor recovery in injured animals [323].

#### 6.5.2. Proteins/Macromolecules (Cytokines, Peptides, Antibodies, Nucleic Acid Cargoes)

Polymer scaffolds have also been used to carry cargoes that target macrophages and influence development of pro-reparative phenotypes [209,310,311]. Lentiviral delivery of IL-10 cytokine is known to induce macrophage polarization towards an M2 phenotype, even in a pro-inflammatory environment [328]. Lentiviral vectors encoding IL-10 alone [209,310] or delivered in combination with other factors such as NT-3 [311] and IL-4 [209] loaded into scaffolds composed of PLGA [209,310,311] and inserted into the hemisected mouse spinal cord [209,310,311], did not appear to impact macrophage numbers but instead skewed the phenotype of cells toward an anti-inflammatory M2-type with an altered macrophage morphology. IL-10 delivery in heparin functionalized PLGA scaffolds significantly increased the number of cells expressing F4/80+ Arginase1+ 14 days after injury, but by 30 days after injury the numbers of Arginase1+ cells reduced and were similar to non-treated controls [310]. These changes were matched by a moderate increase in locomotor performance with similar improvements demonstrated in other studies [209,311,329]. 

#### 6.5.3. Cellular Therapeutics

Incorporating exogenous stem cells into implantable biomaterials has shown particular promise for CNS repair [307,330,331] and the secretomes [332] of stem cells, such as neural precursor cells (NPCs) and mesenchymal stem cells (MSCs), contain a number of different factors capable of modulating the immune response, including macrophage phenotype [333,334].

Biomaterial mediated delivery of MSCs exhibits trophic axon growth promoting properties [268,335,336] and some restoration of function [335] (Table 7). Injection of bone marrow derived MSCs into the contused rat spinal cord without scaffold support resulted in a near four-fold reduction of iNOS+, CD16+/32+ staining of M1 type cells 7 days after injury, with increased expression of arginase-1+ and CD206+ cells in the lesion site, which was associated with increased functional return [337]. Similar changes in macrophage phenotype were noted with umbilical cord MSCs loaded into polyethylene glycol/carbomer and bone marrow MSCs loaded into polyurethane-based reverse thermal gel scaffolds and implanted into the contused mouse spinal cord [218,338].

Adult human bone marrow MSCs implanted into gelatin sponge scaffolds sheathed with a thin film of PLGA and inserted into the fully transected and excised (1.5 mm segment) rat spinal cord resulted in a three-fold decrease in CD68+ cells in the graft and a lower number of CD68+ cells surrounding the scaffold in the lesion site 1 week post-injury [243]. However, further evaluation was not carried out to determine particular subtypes. Similar reductions in infiltrating macrophages were noted after MSCs were implanted in PLGA scaffolds [339] or injected [341]. In a study using human derived MSCs [339] the reduction in macrophage numbers in the PLGA implant was accompanied by increased staining of CD68+ anginase-1+ cells; however, this did not appear to be quantified. Accompanying the reduction in the number of macrophages surrounding injected MSCs, Watanabe and colleagues noted significantly reduced levels of p-p38 mitogen-activated protein kinase and extracellular signal-regulated kinase (p-ERK1/2) in both macrophages and resident microglia, indicating reduced activation of both cell types [341]. Multipotent adult progenitor cells (MAPCs), a subset of stem cells isolated from bone marrow, also exhibit anti-inflammatory properties [342]. Using an in vitro model of neuronal injury MAPCs simultaneously prevented macrophage-induced axonal dieback, reduced matrix metalloproteinase-9 induction and shifted macrophage phenotype from M1 to M2 [342].

Neural stem and progenitor cells (NSPCs) also exhibit immunomodulatory capabilities and a number of studies have demonstrated changes in macrophage responses after implantation [340,343,344,345]. Using a mouse model of contusion injury, Nishimura and colleagues implanted NPCs via syringe at 7 and 42 days after injury to assess the effect of acute versus chronic implantation on cord repair. NSPCs implanted on day 7 but not on day 42 induced significantly greater changes in gene expression of arginase-1 and co-localization of arginase-1 and CD68 in cells; in contrast, no change in expression levels of pro-inflammatory genes were observed in M1 macrophages [345]. Similar striking changes in M1 phenotype (four-fold reduction compared to control groups) were noted in another study using the same model of injury in the mouse [343] and with NPCs implanted 7 days post-injury; however, there was no change in the relative proportion of CD206+ /CD11c (M2) macrophages. In another chronically injured spinal cord model, Riemann and colleagues noted significantly fewer pro-inflammatory M1 macrophages but no change in anti-inflammatory M2 macrophages in the lesion cavity 8 weeks after injury, indicating that NPCs did not encourage an increase in M2 macrophages [344]. These data indicate that implanted NSPCs possess lower efficacy to modulate the chronic M1 macrophage driven inflammatory response at later stages (>day 14) of injury. However, it must also be noted that a fibroglial scar has begun developing in the lesion site by this stage with a well-formed glia limitans and associated basal lamina, which may attenuate NPC efficacy [346]. 

NSPCs implanted in scaffolds such as methacrylamide chitosan (MAC) hydrogel protected by an outer chitosan conduit may modulate macrophage behavior, reduce ED-1+ cells in the lesion site and improve neuronal regrowth 8 weeks after hemisection injury [340]. Of the limited number of studies that have investigated the changes in macrophage number and phenotype following stem cell loaded scaffolds, there is a marked consistency in the changes seen when compared to studies that injected ‘free’ cells into the lesion site, with fewer macrophages populating the scaffold and surrounding tissue. These studies also suggest that MSCs may encourage greater M2 macrophage numbers in the injury site over longer periods (>14 days) but further studies are clearly warranted.

## 7. Future Directions

Over the past two decades, the development of biomaterials to repair traumatic nervous system injury has increased in complexity as understanding of the multi-faceted nature of neuronal repair grows. In particular, the identification of the key role played by macrophages in the nerve micro-environment has given rise to the emerging field of immune-modulatory biomaterials.

Attenuating the macrophage-mediated inflammatory response driven primarily by M1 macrophage phenotypes may have significant benefits, specifically, increasing axonal regrowth [80,111]. Of particular interest are studies that demonstrate the effect of biomaterials’ stiffness, and the macro- and micro-scale architectural features that intrinsically influence macrophage phenotype. As a result of the advent of novel fabrication techniques such as 3D printing, these micron/nano-level modifications are increasingly attainable. The arrival of more controlled fabrication has never been more timely, because we consider that any future, clinically successful immuno-modulatory biomaterial will likely require some or all of these surface modifications. At a base level, implants need to be immune inert. This is particularly the case given the issues revolving around chronic macrophage polarization and the foreign body response we describe in the clinically approved peripheral nerve guidance conduits.

Furthermore, the ability to incorporate therapeutic immunomodulatory cargoes (e.g., small molecule drugs, proteins, oligonucleotides and stem/precursor cells) has prompted the development of multi-component scaffolds/hydrogels. This offers the potential to develop temporally encoded sequential or sustained release of therapeutic agents that influence macrophage phenotype at different stages in the repair process. Although promising, there are several caveats for future work aimed at translation of controlled release strategies for macrophage modulation; namely, they will need to ensure the stability and reliability of any controlled release system. Any strategy that inadvertently exerts too rapid a change in macrophage number and/or polarization may prove counterproductive and impede the neuronal repair process. Immunomodulation is not the elimination of the M1 phenotype, but rather ensures the timely transition to M2 pro-repair phenotypes occurs at the right time and with sufficient effect to achieve a meaningful improvement in axonal regrowth. Care must be taken that introduced nucleic acids, proteins and cells do not result in their own immune reaction and induce or exacerbate chronic inflammation. Similarly, the degradation products or leachables of any particulate delivery system should not result in their own inflammatory reaction.

On a more practical level, it is important that efforts are made for future studies to have clinical relevance. Current pre-clinical work almost always assumes biomaterial implantation immediately following PNS or CNS injury. However, in most cases, medical stabilization of the patient takes precedence, with surgical intervention usually occurring in the days to weeks after injury. Therefore, the biomaterial implant will encounter an entirely different cellular milieu with macrophage-mediated responses in transition from the initial inflammatory phase to the later proliferative and remodeling phases. For the future, it will be essential to verify that biomaterial implants retain immunomodulatory functionality at different stages of nervous tissue repair. An additional practical consideration is to ensure pre-clinical and clinical studies are comprehensive. There are known sex-based differences brought about by the anti-inflammatory effects on macrophages of estrogen, progesterone and allopregnanolone in PNS and CNS [347,348]. Failure to account for these differences will result in a sub-optimal therapeutic intervention. In conjunction with sex-based differences, the age of the patient population should always be considered. For example, resident microglia show morphologic changes during brain development and can be considered phenotypically different between the adult and neonatal brain [349]. 

Regarding CNS injuries, it has been shown that there are differences in terms of immune and inflammatory response between neonatal and adult brains. In particular, resident microglia are the main responding cells in adult injuries, whereas peripheral derived macrophages are predominant in injuries in developing brains [350]. Furthermore, Chhor et al. reported that neonatal resident microglia can have a beneficial role following traumatic brain injury, by expressing markers relative to the regenerative phenotype [351]. For hypoxia-ischemia, resident microglia remain increased longer in the adult hippocampus, but are activated faster in the neonatal, and here are associated with a stronger inflammatory response, as seen by a higher expression of galectin-3 [352].

Fundamentally, there remains a multitude of questions to be answered regarding the macrophage phenotype and its impact on neuronal regeneration. For example, do all M1 macrophages transition to the M2 phenotype of which they are capable? Furthermore, it will be necessary to determine within the injured tissue if all M2 subtypes develop from a common M2 progenitor or have a more direct activation pathway from an M1 subtype. Possessing a clearer understanding of macrophage activation pathways and where they develop in relation to repairing tissue structures will be key for future biomaterial designs. Evidence from several studies already indicates that biomaterial implants may affect the regional distribution of M1/M2 macrophages with greater numbers of M2 macrophages present within the internal structures of implants and with M1-type cells restricted to the periphery [288]. These findings may indicate a complex interplay between macrophage behavior and the implanted biomaterial. For the future, studies that attempt to resolve the spatial and temporal distribution of macrophage subtypes with biomaterial scaffolds/hydrogels in the first days after implantation will undoubtedly provide greater insights into their immunomodulatory function(s).

## 8. Conclusions

The pivotal role macrophages play in coordinating and regulating the inflammatory response to injury in the central and peripheral nervous systems has received much warranted attention over the past 20 years. It is now clear that macrophage-driven responses to acute injury and their synergistic interaction with glial cells at the site of injury are critical for host tissue driven inflammatory responses in both the CNS and PNS. Enhancing the necessary transition from the inflammation associated M1 phenotype to the tissue remodeling associated M2 phenotype, a key feature of peripheral nerve repair and one lacking from the CNS injury response, may drive a stronger regenerative response. Biomaterials offer considerable potential to help influence and enhance the M1/M2 transition in lesion sites through their composition and physiochemical properties, in addition to their ability to carry multiple different cargo types. Building on recent advances, coupled with careful tailoring of composite materials, internal architecture design and functionalization with various cargo types, will lead to future generations of biomaterial implants capable of exerting fine spatiotemporal control of macrophage behavior and drive better regenerative outcomes. 

## Figures and Tables

**Table 1:** Physical modifications for the polarization of macrophages during PNI.

TECHNIQUE/PRODUCT			
Physical Approach	In vitro/In vivo	Immune Response	Ref
**Micropatterned substrates containing fibronectin and Pluronics F127**			
Micropatterns	BMDMs C57Bl/6J mice	Elongated macrophages on micropatterns express M2 phenotype biomarkers	[176]
**(P(LLA-CL)) nerve guidance conduit**			
Aligned nanofibers	* Sciatic nerve from SD rats	Higher expression of arg1, IL-10 and higher M2/M1 ratio compared to random nanofibers	[177]
**Microstructured PFPE surfaces**			
Micropatterns	PBMCs human macrophages	More M2 macrophages expressed in post patterns than line patterns and greater distance between posts induce a stronger anti-inflammatory effect	[178]
**PLCL nerve guidance conduit**			
Micropatterns and GO nanosheets	* Sciatic nerve in SD rats	Micropatterned and GO-modified conduit promotes M2 phenotype differentiation	[173]
**PCL macroporous nanofiber wrap**			
Pores and fibers size	Sciatic nerve SD rats	Macroporous nanofiber wrap increases IL-10 expression and decreases TNF-α expression and M1/M2 ratio on the injury site	[172]

Annotation: Bone marrow derived macrophages (BMDMs); poly(l-lactide-*co*-caprolactone) (P(LLA-CL)); peripheral blood mononuclear cells (PBMCs); Sprague Dawley^®^ (SD); graphene oxide (GO); Arginase-1 (arg1); interleukin-10 (IL-10); Tumour necrosis factor alpha (TNF-α); * In vitro and in vivo.

**Table 2:** Chemical modifications for the polarization of macrophages during PNI.

TECHNIQUE/PRODUCT			
Chemical Approach	In vitro/In vivo	Immune Response	Ref
**Collagen-based nerve guidance conduit**			
Genipin crosslinking	THP-1 cells	Crosslinking of outer conduit reduced M1 polarization	[188]
**Graphene—based conductive fibrous scaffold**			
Graphene modification and electrical stimulation	* Sciatic nerve 4-week-old SD rats	Induced change in macrophages phenotype from M1 to M2	[189]
**Graphene crosslinked collagen-based nerve conduit**			
Graphene modification	Raw 264.7 macrophages	Macrophages seeded onto graphene crosslinked conduit show high expression of CD163 and CD206 markers	[190]
**Microporous nanodiamonds/PCL nerve bridge**			
Nanodiamond addition	Sciatic nerve in male SD rats	Nanodiamonds induce M2 polarization of macrophages	[191]

**Annotation:** Sprague Dawley^®^ (SD); * In vitro and in vivo.

**Table 4:** Organic and synthetic constructs used to induce macrophage polarization in central nerve injury. Where ↑ indicates an increase in specific cells population and ↓ indicates a decrease in specific cell population.

Biomaterial	Model	Level	Delivery	Duration	Therapeutic	Effect	Ref
Peripheral Nerve (Decellularized hydrogel)	SCI Rat (Sprague Dawley)	C3/C4 Unilateral contusion	Injection	14 days	none	No change M1 CCR7+ cells ↑M2 CD206+ cells	[211]
Brain, cord and urinary bladder (Decellularized hydrogel)	SCI Rat (Wistar)	T8 2 mm hemisected excision	Injection	56 days	none	Spatial differences in CD86^+^ (M1) and CD206^+^ (M2) cells ↑ argninase-1 expression	[288]
Urinary bladder (Decellularized hydrogel)	Rat (Sprague Dawley)	Middle cerebral artery occlusion Cortex	Injection	14 days	none	No change CD86^+^ (M1) cells CD206^+^ (M2) Present in hydrogel	[212]
(Decellularized hydrogel)	Rat (Sprague Dawley)	T9-10 200 kdyne Compression (Moderate- severe injury)	Injection	56 days	none	↓ total ED1+ cells ↓ED-1+, CD86+ (M1) cells ↑ ED-1+, Arginase-1+ (M2) cells ↑ Expression CD206, arginase1, and IL-10	[241]
Collagen	SCI Rat (Sprague Dawley)	T7-11 2 mm Lateral hemisected excision	Scaffold	28 days	MSCs	↓total CD68+ macrophages ↑ CD206, arginase-1, and IL-10 expression	[240]
Gelatin	SCI Rat (Sprague Dawley)	T10-11	Scaffold	56 days	MSCs	↓total CD68+ macrophages ↓ IL-1β and TNF-α expression	[243]
HA combined with PCL spun fibers	SCI Rat (Sprague Dawley)	T9 175 kdyne compression (Moderate injury)	Scaffold	28 days	none	No change CD86^+^ (M1) cells ↑M2 CD206+ (M2) cells	[255]
Agarose/polyethylene glycol/carbomer	SCI (C57BL/6J mice	Aneurysm clip compression	Scaffold	9 days	MSCs	↑TNF-α (M1) and 10-fold ↑ arginase-1 expression	[218]
Chitosan and water as fragmented physical hydrogel suspension	Rat (Wistar)	Bilateral dorsal over-hemisection	Injection	56 days	none	↓ CD86+ (M1) expression ↑M2 CD206+ (M2) expression	[213]

**Table 5:** Chemical methods that modify macrophage polarization in CNS injury. Where ↑ indicates an increase in specific cells population and ↓ indicates a decrease in specific cell population.

Biomaterial	Model	Level	Delivery	Duration	Therapeutic	Effect	Ref
Oligo[poly(ethylene glycol) fumarate (OPF) plus Matrigel	SCI Rat (Sprague Dawley)	T9-10 Complete transection	Scaffold	56 days	Schwann cells	No change CD86^+^ (M1) cells ↑M2 CD206+ (M2) cells	[208]
Poly(glycolic acid) (PGA)	SCI Rat (Sprague Dawley)	T10-11 3 mm Lateral hemisected excision	Scaffold	56 days	NPCs	↓CD68+ macrophages ↑M2 CD206+ (M2) cells	[273]
PLGA and Poly-L-lysine	SCI Rat (Sprague Dawley)	T10 220 kdyne contusion (Moderate-severe injury)	Scaffold	84 days	none	↓CD74 + (M1) cells ↑CD206+ CD163+ and Arginase-1+ (M2) cells	[274]
GO	Rat (Wistar)	C6 2 mm lateral hemisected excision	Scaffold	10/30 days	none	Day 10: No change CD80+ (M1) and CD163+ (M2) cells Day 30: ↓CD86+ (M1) cells Trend ↑ CD163+ (M2) cells	[308,309]
PLGA	mice (C57/BL6)	T9-T10 2.25 mm lateral hemisected excision	Scaffold	30 days	none	No change F4/80+ arginase1- (M1) cells ↑ F4/80+ arginase 1+ (M2) cells	[310]
PLGA	mice (C57/BL6)	T9-T10 2 mm lateral hemisected excision	Scaffold	28 days	none	↓ CD86, MHCII, iNOS (M1) expression ↑CD206, (Retnla), arginase-1 (M2) expression ↑Hoechst+/F4/80+/Arginase-1+ (M2) cells	[209]
PLGA	mice (C57/BL6)	C5 1.15 mm lateral hemisected excision	Scaffold	84 days	none	↑F4/80+/Arginase-1+ (M2) cells	[311]

**Table 6:** Nanoparticle formulation methods for modifying macrophage polarization in central nerve injury. Where ↑ indicates an increase in specific cells population and ↓ indicates a decrease in specific cell population.

Biomaterial	Model	Level	Delivery	Duration	Therapeutic	Effect	Ref
Chitosan	SCI Rat (Sprague Dawley)	C3-C4 1–2 mm lateral hemisected excision	Spongostan^®^	7 days	None	↓ ED-1+ macrophages	[299]
Minocycline loaded poly-caprolactone nanoparticles	Mice (C57/BL6)	T12 Unilateral stab/ injection	Cord Injection	15 days	None	↓ CD68+ macrophages	[298]
Flavopiridol loaded PLGA nanoparticles	SCI Rat (Sprague Dawley)	T9-10 Lateral hemisection	Topical delivery	42 days	None	↓CD68+ macrophages ↓IL-6 and IL-1β and increased ↑IL-10 expression	[323]
Hirudin loaded Pluronic F-127 hydrogels	mice	T8 Contusion with 0.5 mm displacement	Cord injection	28 days	none	↓CD68+ macrophages	[301]
PLGA nanoparticles	mice (C57/BL6)	T9-10 1.2 mm lateral hemisected excision	PLGA Scaffold	84 days	none	↓ iNOS+, CD86+, and MCP-1+(M1) expression ↑ CD206+ and IL-10+ (M2) expression	[324]
Minocycline hydrochloride complexed with polysaccharide dextran sulphate	SCI Rat (Sprague Dawley)	C5 200 kdyne contusion (Moderate to severe injury)	Agarose Scaffold and intraperitoneal injection	42 days	none	↓CD68+ macrophages ↓ ratio iNOS+, CD68+ (M1) cells to arginase 1+, ↑CD68+ (M2) cells	[297]
Chicago sky blue loaded liposomes	SCI Rat (Sprague Dawley)	T9 150 kdyne contusion (Moderate injury)	Intravenous injection	4 days	none	↑ CCL2, IL-1β, and iNOS (M1) expression ↑ arginase I and TGF-β (M2) expression ↑ arginase I+, CD68+ (M2) cells	[326]

**Table 7:** Summary of the different types of stem cell used to treat CNS injury and their effect on macrophage number and phenotype in the lesion site. Where ↑ indicates an increase in specific cells population and ↓ indicates a decrease in specific cell population.

Biomaterial	Model	Level	Delivery	Duration	Therapeutic	Effect	Ref
None Cell suspension	SCI Rat (Sprague Dawley)	T9-10 200 kdyne contusion (Moderate to severe injury)	Injection	35 days	MSCs	↓ iNOS+, CD16+/32+ (M1) cells ↑ arginase 1+, CD206+(M2) cells	[337]
Gelatin sponge scaffolds sheathed with PLGA film	SCI Rat (Sprague Dawley)	T10-11 Full transection	Scaffold	56 days	MSCs	↓ CD68+ macrophages ↓ CD68+, IL-1β (M1) cells ↓ CD68+, TNF-α (M2) cells	[243]
PLGA	SCI Rat (Sprague Dawley)	T9-10 4 mm lateral hemisected excision	Scaffold	10 days	MSCs	↓ CD68+ macrophages ↑CD68+ anginase-1+ (M2) cells	[339]
Methacrylamide chitosan hydrogel	Rats (Fischer 344)	T8-9 2.0–2.5 mm lateral hemisected excision	Scaffold	56 days	NPSCs	↓ ED-1+ macrophages	[340]
